# Recent Advances in the Chromatographic Analysis of Emerging Pollutants in Dairy Milk: A Review (2018–2023)

**DOI:** 10.3390/molecules29061296

**Published:** 2024-03-14

**Authors:** Dina Ashraf, Rana Morsi, Muhammad Usman, Mohammed A. Meetani

**Affiliations:** Chemistry Department, College of Science, United Arab Emirates University, Al-Ain P.O. Box 15551, United Arab Emirates; 201350165@uaeu.ac.ae (D.A.); 201350167@uaeu.ac.ae (R.M.); 700039308@uaeu.ac.ae (M.U.)

**Keywords:** emerging pollutants, dairy milk, gas chromatography, liquid chromatography, extraction

## Abstract

Emerging pollutants (EPs) encompass natural or synthetic substances found in the environment that pose potential risks, but which have only recently been recognized or monitored. EPs consist of various categories, including pesticides, pharmaceuticals, hormones, mycotoxins, and endocrine-disrupting chemicals (EDCs). Through several pathways, EPs can access food, potentially leading to health impacts when safe concentrations are exceeded. Milk, being a highly nutritious food product that is heavily consumed by many consumers of different ages, is a crucial food matrix where EPs should be regularly monitored. In the literature, a large number of studies have been dedicated to the determination of different EPs in dairy milk, employing different analytical techniques to do so. Chromatography-based techniques are the most prevalent means used for the analysis of EPs in milk, demonstrating significant efficiency, sensitivity, and accuracy for this specific purpose. The extraction of EPs from a complex matrix like milk is essential prior to performing chromatographic analysis. This review comprehensively covers relevant research papers on the extraction and subsequent detection and determination of EPs in milk using chromatographic methods from 2018 to 2023.

## 1. Introduction

In recent years, the global population has witnessed rapid growth, leading to a surge in consumer demand. This increase has resulted in the expansion of industrial manufacturing, agricultural activities, and technological development. Consequently, both the environment and humans have been exposed to various new chemicals known as emerging pollutants (EPs) or contaminants of emerging concern (CECs) [1]. EPs are defined as synthetic or naturally occurring compounds found in the environment. They are generally not monitored, but have the potential to cause adverse ecological effects and health consequences [2,3]. EPs can broadly be divided into three chemical categories: the first encompasses newly synthesized compounds; the second includes compounds that have long been present in the environment but which have only recently been detected and recognized; and the third comprises compounds that have been known for some time, but whose detrimental effects on the environment and human health have been identified only recently [2,4].

EPs consist of a wide array of organic and inorganic compounds commonly found in the environment, such as pesticides, perfluorinated compounds, pharmaceuticals, personal care products, endocrine disruptors, hormones, toxins, plasticizers, flame retardants, and more [1,4]. The majority of EPs stem from routine anthropogenic activities, including domestic, healthcare, agricultural, and industrial processes [5]. These substances can infiltrate the environment, permeating various food sources and environmental matrices, such as water, soil, marine sediments, and both indoor and outdoor dust [1]. The global production of these pollutants is estimated to have surged from 1 million to 500 million tons annually [6]. EPs are recognized as potential environmental hazards due to their high toxicity and biochemical reactivity. They have adverse effects on the quality of natural resources and pose significant health risks to humans and other living organisms [7]. Many of these pollutants persist in the environment and tend to bioaccumulate in animal tissues [1]. Additionally, a significant number of them can be transported over long distances in the environment [8]. Consequently, assessing the health risks associated with human exposure to these contaminants becomes paramount. There are several pathways through which individuals can be exposed to EPs: the inhalation of volatile EPs present in the air, direct skin contact, and ingestion. Factors such as the amount, frequency, and duration of exposure play a critical role in determining the risks associated with these pollutants. Furthermore, individual factors, such as diet, sex, age, lifestyle, and genetic makeup, can significantly influence susceptibility to their effects [1,8].

EPs pose a spectrum of health risks to humans, ranging from mild symptoms such as headache, dizziness, nausea, and skin irritation to severe conditions including cancer, reproductive disorders, heart diseases, nervous system disorders, liver damage, DNA mutation, among others [9]. For example, a study conducted by Bonefeld-Jorgensen et al. found compounds in serum and a strong correlation between the presence of perfluorinated compounds and an increased risk of breast cancer in Inuit women from Greenland [10]. In another research study, a significant relationship was identified between elevated levels of certain liver enzymes (alkaline phosphatase, gamma-glutamyltransferase, and lactate dehydrogenase) and bisphenol A, an endocrine disruptor, indicating a potential alteration in liver function [11].

**Figure 1:**
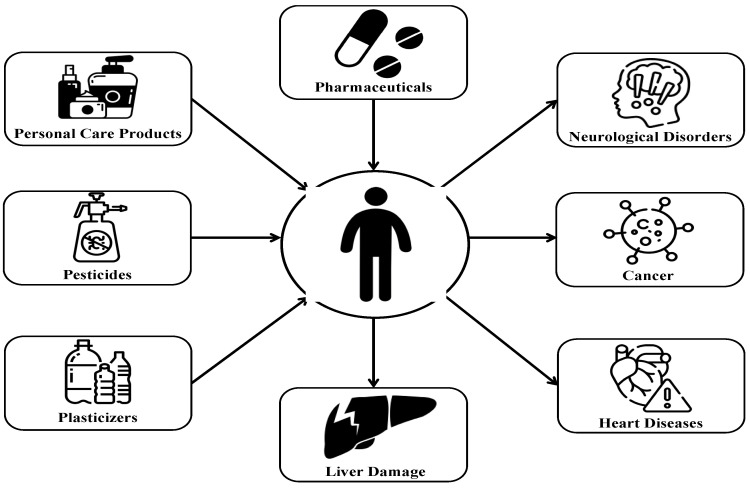
Health impacts of different types of EPs.

Figure 1 is a graphical representation of some potential health risks posed by different types of EPs, as demonstrated in the literature.

Notably, the adverse effects of EPs are not limited to humans. Evans et al. examined the impact of endocrine disruptors in Canada’s Oldman River waters on the gene expression of the Longnose Dace fish species [12]. With approximately one-third of the 28,000 km^2^ watershed allocated to agricultural activities, particularly to intensive livestock operations, runoff introduces significant amounts of endocrine disruptors into the river. Consequently, this causes alterations in the fish’s gene expression, notably leading to the feminization of male specimens. Given the potential risks posed by these pollutants to both humans and the environment, numerous studies have been dedicated to providing comprehensive insights into their occurrence, potential impacts and fate, and also to developing analytical methods for their detection in various food and environmental matrices [13,14,15,16].

## 2. Emerging Pollutants in Dairy Milk: A Concern for Public Health

Among various matrices containing EPs, dairy milk has emerged as a critical focal point. Renowned for its exceptional nutritional benefits, milk ranks among the most consumed foods worldwide. It is a vital reservoir of protein, essential nutrients such as calcium, phosphorus, magnesium, zinc, iodine, and potassium, and essential vitamins including A, D, B12, and B2 [17]. Due to its rich nutritional profile, milk plays a fundamental role in the diets of infants and young children. However, various EPs, including veterinary drugs, antibiotics, endocrine disruptors, phthalates, pesticides, and others, can contaminate milk [15,18,19,20]. The Food and Agriculture Organization of the United Nations has reported the distribution of the average consumption of milk in different areas of the world. The data are based on per capita food supply at the consumer level. For the year 2020, they reported some nations that consume less than 50 kg of milk per year, such as China, India, and Iran, and other nations consuming up to 290 kg of milk per year, such as Albania, Switzerland, and Kazakhstan [21].

The presence of EPs in dairy milk can arise from multiple sources: contaminated cattle feed, polluted water sources, and residues from veterinary medicines. Notably, pesticide residues can find their way into animal feed due to improper application in agricultural practices. Moreover, milk’s fat content makes an ideal medium for dissolving lipophilic pesticides [21,22]. Contamination may also occur when using polluted water to clean equipment involved in milk storage and processing, or when providing such water as drinking water for cattle [23]. Additionally, the use of veterinary drugs and antibiotics in cattle for disease prevention and treatment can introduce drug residues into milk [19,24]. During the mechanical milking process, transportation from the farm’s cooling tanks to the dairy factory’s cooling tank, and packaging, phthalates might migrate into the milk [25].

While these contaminants may exist in minute quantities, they still pose serious health risks, especially if they exhibit persistence and bioaccumulative properties [1]. The regular or daily consumption of milk means that even trace amounts of these contaminants can accumulate significantly over time, posing a threat to consumer health. This concern is particularly critical for infants and children given their heightened vulnerability due to their ongoing physiological development. Therefore, the thorough evaluation of milk quality is essential to ensure food safety and reduce the health risks associated with these contaminants. Several studies have highlighted the detection of various EPs in milk, emphasizing the importance of using comprehensive monitoring systems for animal feed, water, and medicines. These findings draw attention to concerns regarding milk’s safety [15,26,27,28]. Numerous studies have developed analytical methods for the evaluation of different types of EPs in milk and milk products. This review comprehensively covers all relevant research papers dedicated to the development of chromatography-based analytical methods for determining different categories of EPs present in dairy milk from 2018 to the present (2023). To maintain focus and coherence in this review, the scope excludes other dairy products, non-dairy or plant-based milk, and human milk due to the extensive volume of research studies available in these areas. Comprehensively covering all these areas in a single review would be impractical.

## 3. Chromatographic Techniques for EP Analysis

In its fundamental concept, chromatography is based on the separation of sample components that have been immobilized on a moving phase (mobile phase) over a fixed phase (stationary phase). The components of different samples interact differently with the stationary phase and hence move slower or faster, spending different times in this phase (retention time) until they elute from the column, which enables their separation. The mobile phase can be either gas or liquid, while the stationary phase can be solid or liquid.

Gas chromatography (GC), utilizing gas as the mobile phase, and liquid chromatography (LC), in which the mobile phase is a liquid, are the most popularly employed types of chromatography for analytical purposes. When combined with different types of detectors, such as mass spectrometers (MSs), ultraviolet detectors (UVs), diode array detectors (DADs), fluorescence detectors (FLDs), flame ionization detectors (FIDs), and electron capture detectors (ECDs), GC and LC play pivotal roles in the analysis, identification, and quantification of a wide variety of contaminants in food and environmental matrices, offering significant efficiency and sensitivity [14,29,30,31]. Delving into the literature makes it evident that the most commonly employed methods for analyzing and quantifying residual contaminants in milk and dairy products generally rely on chromatographic techniques [13,32,33,34].

### 3.1. LC-Based Techniques

Paired with different detectors, typically FLD, UV, DAD and MS, LC-based techniques emerge as a robust and powerful option for the analysis of a wide range of compounds with different chemical and physical properties.

For decades, LC-MS has found applications in the separation and determination of various contaminants in complex food and environmental matrices [32,35,36,37]. In this system, as the separated analytes elute from the column, they are introduced into the mass spectrometer, in which they are accelerated through magnetic and electric fields. This leads to their separation based on their mass-to-charge ratio (*m*/*z*), providing information about their identity as well as their quantity. Moreover, the availability of different types of mass spectrometers, each differing in their ionization sources and/or mass analyzers, has further expanded the range of compounds that can accurately be detected. These mass analyzers include time-of-flight (TOF) devices, Orbitrap, and tandem mass spectrometers.

Tandem mass spectrometry (MS/MS) or (MS2), which can be seen as an extension of MS, involves the use of two sequential mass spectrometry stages, allowing for the detection of trace amounts of analytes with superior sensitivity. MS/MS, in combination with LC techniques such as high-performance liquid chromatography (HPLC) and ultra-high-performance liquid chromatography (UHPLC), is the most prevalent chromatographic method used for the analysis of residues of different categories of EPs in milk. These include veterinary drug residues, pesticides, endocrine-disrupting compounds (EDCs), and others [32,38,39,40]. For example, Nemati et al. employed HPLC-MS/MS for the determination of residues of seven different pesticides in cow milk, with the limit of detection (LOD) ranging between 0.09 and 0.27 ng/mL [41]. Moreover, a method of analysis was developed based on UHPLC-MS/MS and validated by Macheka et al. for use in the determination of compounds from the category of per- and polyfluoroalkyl substances (PFASs) in dairy milk and infant formula with low LOD values within the range of 0.005–0.05 ng/mL [42]. Guedes-Alonso et al. also successfully applied another method based on UHPLC-MS/MS for the detection of fifteen steroid hormones in commercial raw milk, achieving low LODs ranging between 0.047 and 1.242 ng/mL [27].

In recent years, the goal of shortening analysis times with increasing sample throughput, sensitivity, and resolution has driven the development of ultrafast separations and high-resolution MS (HRMS) detectors. Wu et al. incorporated the separation capabilities of liquid chromatography with the accurate identification and detection of high-resolution mass spectrometry (LC-HRMS) for the determination of eight peptide antibiotics in three different types of bovine milk, with LODs ranging between 0.5 and 5.5 ng/g. These values are far below the limits of concern for these types of antibiotics [43]. Similarly, LC-HRMS was also the technique of choice by Wang et al. for the selective and sensitive analysis of two antibacterial drugs, vancomycin and norvancomycin, in milk samples, with LODs of 0.15 μg/kg for both [44].

In addition to MS, fluorescence detectors offered high efficiency and precision at specific degrees of excitation and at certain emission wavelengths. These qualities were in many cases comparable to those of MS. For naturally fluorescent analytes or analytes that can be altered to become fluorescent, coupling HPLC with a fluorescence detector (HPLC-FLD) is a particularly valuable analytical method. Badali et al. proposed a method utilizing HPLC-FLD for the determination of two types of poisonous mycotoxins that are produced by certain molds, namely, aflatoxin M1 (AFM1) and ochratoxin A (OTA) [45]. The developed method achieved low LODs of 0.37 and 0.25 and ng/L for AFM1 and OTA, respectively. This method was applied for the detection of the two analytes in samples of cow milk. Similarly, Murshed employed HPLC-FLD for the determination of AFM1 in milk and milk products including powdered milk and yogurt, achieving an LOD of 0.002 μg/L [46]. While HPLC-FLD was the method of preference for the analysis of mycotoxins in milk, it was rarely employed for the determination of other types of EPs, such as veterinary drugs and pesticides.

The application of liquid chromatography in conjunction with UV detectors was one of the earliest methods used for different purposes such as the detection and quantification of different categories of food and environmental pollutant residues [47,48,49]. However, UV-Vis detectors in which the detection and identification of the eluants are based on their absorption into the UV or visible region of the electromagnetic spectrum suffer from major drawbacks. These include avoiding the use of variety of solvents that absorb strongly in the UV region, such as ethyl ethers, chloroform, acetone, and benzene, due to their interference with the target analytes [50]. Even a common solvent like methanol absorbs in the UV region, despite being used in the mobile phase for HPLC-UV, but precautionary steps and gradient elution are important in order to suppress its interference. This limitation of solvent options subsequently narrows and restricts the applicability of HPLC-UV systems. Moreover, it is not possible to assess compounds that do not contain chromophores (the functional groups that are absorbed in the UV-Vis region) using this system without a derivatization step. This in turn consumes large amounts of sample, solvents, and hazardous chemicals, in addition to lengthening the duration of the procedure, adding complexity to the analytical method, and demonstrating another major drawback of HPLC-UV systems.

Despite being surpassed by MS and FLD detectors, the combination of UV with HPLC still finds applications in the analysis of many classes of pollutants in milk. For example, Al-Afy et al. monitored tetracycline (TCN), oxytetracycline (OTC), and doxycycline (DC) antibiotics, which belong to the family of broad-spectrum tetracycline (TCN) antibiotics in bovine milk, by using an analytical method based on HPLC-UV for their separation and detection [51]. The LOD was obtained within the range of 1.8–2.9 μg/L. HPLC with diode array detection (HPLC-DAD), which is also referred to as photodiode array (PDA) detector (HPLC-PDA) analysis, is a method in which the absorbance of compounds is measured over a wide range of wavelengths in the UV-Vis region at one time (simultaneously), providing more detailed spectral information. This allows for more precise and accurate compound identification, and it is also a powerful technique that has been used in the context of EP determination in milk. An example is the method provided by Vuran et al. for the determination of two antibiotics in milk samples: chloramphenicol and tetracycline. LODs were 3.43 ng/mL and 3.55 ng/mL and the method was validated for its applicability in the analysis of these compounds in complex matrices like milk [52].

While HPLC and UHPLC systems, coupled with the aforementioned detectors, currently dominate the analysis of EPs, recent efforts have been made to find alternative approaches that are less time-consuming, less complex, more cost-effective, and more environmentally friendly [53,54]. Such approaches include the employment of capillary liquid chromatography (CLC) and micellar liquid chromatography (MLC) [54,55]. Tejada-Casado et al. implemented CLC in conjunction with a UV detector for the determination of sixteen different anthelmintics drugs from the benzimidazole group in milk [55]. This method achieved low LODs ranging between 1.0 and 2.8 μg/kg, providing an efficient and miniaturized chromatographic trial for the purpose of determining the presence of EPs in milk. This technique was also reported to be simpler and greener owing to reduced solvent and sample consumption. Similarly, Prasad Pawar et al. proposed a simple, cost-effective, and environmentally benign approach that used MLC for the assessment of residues of mebendazole anthelmintic drugs in samples of milk and dairy products, as well as breeding waste from bovine animals [54]. This method demonstrated good sensitivity, which was reflected in the low LOD ranging from 0.1 to 0.2 ppm. These studies highlighted the potential of using simple liquid chromatographic techniques as alternatives for conventional HPLC- and UHPLC-based methods that, despite being powerful and sensitive, still require expertise, involve time-consuming preconcentration steps, and are comparatively much more expensive.

### 3.2. GC-Based Techniques

Chromatographic methods based on GC, including GC-MS, GC-FID and GC-ECD, have been reported by numerous studies to demonstrate high efficiency, sensitivity, selectivity, and precision in the determination of various categories of complex contaminants in milk [37,56,57].

In most studies of EPs in milk samples, GC equipped with single MS provided better results than studies using GC with other detection systems, such as FID and ECD. Yet, tandem mass spectrometry (MS/MS) has been applied in recent years to further improve precision and sensitivity.

Using the high separation capability of GC in combination with the efficient detection of MS, Campos do Lago et al. proposed a method for the determination of four organophosphates pesticides, with LODs ranging from 0.36 to 0.95 μg/L [58]. This method was efficiently applied for the detection of these pesticides in commercial bovine milk samples. Bisphenol A and five phthalate esters were targeted by Tang et al., who developed an analytical method, also based on GC-MS, which achieved low LODs within the range from 0.01 to 0.06 μg/L [59]. While Pan et al. has employed GC-MS/MS for developing a valid method for determination of six phthalate esters, achieving LODs ranging from 0.8 to 2.1 μg/L [60]. This method was suitable for the investigation of targeted phthalates in milk samples. GC-MS/MS was also the technique of choice for Hasan et al., who targeted a group of compounds under the two categories of polychlorinated biphenyls (PCBs) and polyaromatic hydrocarbons (PAHs) in a total of 100 cow milk samples [34]. This method achieved low LOD values ranging from 0.016 to 0.031 ng/g for the targeted PCBs and from 0.3 to 1.0 ng/g for PAHs.

A flame ionization detector (FID) is a unique type of detector in which the sample is burned in a flame, which in turn generates electrically charged ions. The electrical current produced by those charged particles is what is measured in FID and it is proportionally related to the quantity of ions. J. Zhang et al. used FID coupled with GC for the determination of eight phenolic compounds, achieving LODs within the range of 0.001–0.1 μg/L under optimum conditions [57]. The method was applied for the determination of those analytes in five types of canned beverages, including milk.

In addition to MS and FID, an electron capture detector (ECD) is a highly sensitive type of detector used alongside GC. ECD is a specialized tool for the detection of electron-absorbing analytes or electronegative compounds that have high affinities to electrons such as chlorinated pesticides, polychlorinated biphenyls (PCBs), and some types of drugs. These types of compounds attract electrons emitted by the radioactive source in an ECD, producing charged species (ions). The amount produced is directly proportional to the concentration of the target analyte. Rahman et al. developed an analytical method based on GC-μECD for the determination of an organochlorine pesticide (endrin) and its metabolite (δ-keto endrin) in five food products of animal origin (chicken, pork, beef, egg, and milk), with an LOD that reached 0.003 mg/kg [61].

It is worth mentioning that different chromatographic techniques, coupled with different types of detectors, are shown to be reliable for the detection of different types of EPs, either specifically or simultaneously. Although the majority of the studies on this issue have provided methods for the detection of compounds that belong to the same category of EPs, there are several studies that have provided chromatographic methods that are valid for the determination of multiclass residues of EPs. Jia et al. developed an analytical method, employing ultra-high-performance liquid chromatography–hybrid quadrupole–linear ion trap mass spectrometry (UHPLC-Qtrap-MS) for the simultaneous analysis of a total of two hundred and nine contaminants that belong to veterinary drugs, mycotoxins, and pesticide categories [13]. The developed method obtained an LOD ranging from 0.01 to 1 μg.kg and was validated and applied for the investigation of contaminants in bovine milk samples.

Similarly, Izzo et al. employed ultra-high-performance liquid chromatography/high-resolution mass spectrometry (UHPLC-Q-Orbitrap HRMS) for the analysis of a group of mycotoxins and pharmaceutically active compounds in milk, with LODs within the range of 0.001 to 0.010 ng/mL [28].

## 4. Extraction of EPs from Milk

Performed prior to chromatographic analysis, sample treatment is a critical step that involves some preparation procedures, including extraction, the preconcentration of compounds of interest, the clean-up of impurities, and homogenization.

In complex matrices like milk, analytes of interest are required to be selectively isolated, purified, and extracted before their introduction into the analytical technique. The extraction step is significantly useful and significantly affects the overall performance of the analytical method, especially since most of these analytes are present in low concentrations.

### 4.1. SPE

The different extraction techniques used for this purpose include solid-phase extraction (SPE) which, since its introduction in 1980s, has been widely employed as a sample preparation approach [62]. SPE involves passing the sample over solid adsorbents/sorbents with selective affinity to the target analyte, which is usually packed in a cartridge or a column. Target contaminants adsorb to the solid phase, whereas undesired components are washed away. SPE’s advantages include its simplicity, ease of automation, and the utilization of various types of adsorbents that are often readily available [63,64]. Since the solid adsorbent is the key factor in SPE approaches, different types of adsorbents have been developed and enhanced over time. Those solid adsorbents include commercially available adsorbents, as well as selectively synthesized adsorbents.

After solvent extraction and centrifugation, Decheng et al. used a commercial SPE cartridge (PRiME HLB) for the purification and extraction of the steroid hormone progesterone and twenty-one veterinary drugs in the class of progestins from milk samples [29]. The recoveries rates of the spiked milk samples were between 80.7% and 108.3%. In addition, bisphenol A (BPA) and bisphenol S (BPS) were extracted from milk samples using C18 SPE cartridges after their sonication and dilution, reaching average recovery rates of 86% ± 3 for BPA and 100% ± 7 for BPS [65]. Although commercial SPE adsorbents are frequently used, they often exhibit the nonselective adsorption of target analytes, which may in turn decrease the yield and efficiency of extraction. To address this issue, wide varieties of SPE adsorbents are being specifically synthesized and tailored for the selective recognition and extraction of target analytes. In this context, molecularly imprinted polymers (MIPs) have become widely popular as solid adsorbents owing to their ease of preparation, structural predictability, cost-effectiveness, specific recognition capability, and broad applicability [64,66]. For the extraction of lincomycin antibiotics from milk samples, Negarian et al. utilized a selective lincomycin core–shell MIP. The authors then performed its analysis using HPLC-UV, which yielded a recovery rate ranging from 80% to 89% [66]. Additionally, X.-C. Huang et al. also applied MIP as a solid adsorbent for the extraction of three endocrine-disrupting chemicals, namely, hexestrol, nonylphenol, and bisphenol A, from lake water and milk samples, resulting in a recovery rate that ranged from 89.9 to 102.5% [35].

Moreover, carbon nanomaterials have gained great popularity as adsorbents in SPE due to their unique qualities, such as high surface area, excellent adsorption capacity, exceptional chemical activity, chemical stability, and ease of surface modification or functionalization [67,68]. These materials include carbon nanotubes (CNTs), including single-walled CNTs (SWCNTs) and multi-walled CNTs (MWCNTs), graphene oxide (GO), and graphene (G). In the context of EPs in milk, Jiang et al. employed educed graphene oxide and gold nanoparticles (rGO/Au) for solid-phase extraction of nine different mycotoxins from milk. The recoveries achieved were in the range of 70.2–111.2% [69]. Conversely, (N. Li, Qiu, et al.) used magnetic MWCNTs modified with polyethyleneimine for the selective extraction of ten different mycotoxins from milk samples before their introduction into the HPLC–MS/MS system [70]. This approach obtained adequate recoveries, ranging from 88.3 to 103.5%. 

### 4.2. MSPE

Magnetic solid-phase extraction (MSPE) is a type of SPE where magnetic sorbents are utilized for target compound extraction and then easily separated, along with the desired analytes, from the sample by simply placing a magnet near the sample. This eliminates the need for time-consuming traditional purification steps like filtration, decantation, or centrifugation.

Many types of adsorbents used for the MSPE of EPs from milk have been reported in the literature. For instance, Guan et al. synthesized core–shell composites of magnetic covalent organic frameworks (COF@Fe_3_O_4_), where the spherical Fe_3_O_4_ was the magnetic core and the COF, which was synthesized via a Schiff base reaction of 1,3,5-triformylphloroglucinol and p-phenylenediamine, was the shell [71]. The synthesized COF@Fe_3_O_4_ was used as an adsorbent for six types of fluoroquinolone antibiotics (enoxacin, fleroxacin, ofloxacin, norfloxacin, pefloxacin, and lomefloxacin) extracted from milk samples after their centrifugation and prior to their introduction into HPLC-UV. High recovery ranges of the spiked six fluoroquinolones were reported in milk samples, ranging from 90.4 to 101.2%.

### 4.3. SPME

Although classical SPE is still commonly applied for sample preparation in conjunction with chromatographic techniques, it has undergone substantial and ongoing advancements over time, offering selective and precise separations at the same time as shortening extraction time by using fewer steps and minimizing hazardous organic solvents. It is not only the development of different types of solid adsorbents, but also the development of new variations of SPE, that allows this method to operate in different modes and formats.

Solid-phase microextraction (SPME), in which a solid microfiber such as a silica rod is coated with an extraction phase selective to the target components, is one example of the advanced variations of SPE in which no or minimized solvents are utilized. SPME is particularly efficient for the extraction of volatile and semi-volatile compounds. It can be carried out by either inserting the SPME fiber directly into the sample or into the headspace (HS) (the gas phase just above the sample). Jeong et al. employed (HS-SPME) for the extraction of the toxic organic compound furan from different food matrices, including milk, peanut butter, tuna, and peanut butter, among others [72]. The extraction fiber was made of 75 μm carboxen/polydimethylsiloxane and the recovery ranged from 77.81 to 11.47% for furan in spiked food matrices.

### 4.4. FPSE

Among the innovative variations of SPE is fabric-phase sorptive extraction (FPSE), which involves the use of a natural or synthetic sorptive fabric that is treated or coated with a selective sorbent material, integrating the principles of both SPE and SPME approaches [73]. The fabric support can be hydrophilic, such as cotton cellulose, or hydrophobic, such as polyesters, or a combination of both depending on the polarity of the target analytes. Different types of sorbents can be bonded to the fabric substrate, such as MIPs or sol–gel adsorbents, depending on the properties of the target compounds that grant this technique’s high selectivity. Moreover, using the fabric substrate as a support for sorbent materials provides them with chemical stability and mechanical robustness [73].

For the extraction of estrogenic endocrine-disrupting chemicals and bisphenol A from milk samples, Mesa et al. used commercial cotton fabric that had been treated and coated with sol–gel adsorbents. They obtained recoveries that ranged from 13.7 to 69.2% and observed that, as the fat content of the milk decreases, the recovery values of the spiked samples increase [14].

### 4.5. IAC

In immunoaffinity columns (IACs), SPE principles are applied through selective antibody–antigen interactions. This extraction approach is commonly applied for the extraction of mycotoxins from food samples prior to their analysis [46,74,75]. For instance, Mannani et al. used IAC for the purification and extraction of AFM1 from milk samples, obtaining mean recoveries ranging between 87% and 95% [75]. Despite the accuracy and selectivity demonstrated by this approach, as reflected in the adequate recovery values, it also suffered from some drawbacks, including its relatively expensive cost and its limitation to a single use [76].

### 4.6. LLE

Liquid–liquid extraction (LLE) is a different type of extraction, which is also known as solvent extraction. LLE along with SPE represent the oldest extraction techniques adopted for the extraction of many contaminants from complex food and environmental matrices [63,77,78].

In LLE, compounds are partitioned between two immiscible aqueous and organic phases. Solvent selection is critical in this type of extraction. Choi et al. applied this technique, using acetic acid in acetonitrile for the extraction of two types of pesticides (tebufenozide and indoxacarb) from different food matrices, including milk, followed by homogenization and centrifugation [79]. The recovery rate ranged between 73.22 and 114.93% in all the studied matrices. Although LLE extraction procedures are frequently applied, they come with several drawbacks, including the consumption of large samples and solvent volumes. These issues contradict the direction of green chemistry’s development, which is low sensitivity, with concerns around possible sample contamination, difficulty of automation, as well as lengthy extraction times.

### 4.7. DLLME, ALLME and SALLE

To overcome those drawbacks, variations of LLE have been developed. These include dispersive liquid–liquid microextraction (DLLME), salting-out-assisted liquid–liquid extraction (SALLE), and air-assisted liquid–liquid microextraction (ALLME). In DLLME, the working mechanism involves a ternary solvent system that consists of a water-miscible solvent (dispersive solvent), water-immiscible solvent (extraction solvent), and an aqueous sample with target analytes. The extraction and dispersive solvents are mixed and rapidly injected into the aqueous sample, forming a cloudy solution in which fine droplets of the extraction solvent are dispersed in the aqueous sample, acting as highly efficient extractors for the target organic compounds. The large contact area between the extraction solvent microdroplets and the aqueous sample provides this extraction approach with high efficiency, rapidity, good recovery, and a high enrichment factor [77,80]. Melamine was extracted from milk via DLLME by Vaseghi Baba et al. before subsequent analysis using HPLC-UV, an extraction method that resulted in satisfactory relative recovery rates ranging from 79.6 to 105.0% [49]. Additionally, a study conducted by Sharma et al. revealed the applicability of DLLME for the extraction of eight pesticides from milk, with a recovery rate within a range from 86.15 to 112.45% [81]. 

In SALLE, a water-miscible organic solvent, such as acetonitrile or methanol, is mixed with the aqueous sample that contains the target compounds. A high concentration of salts, such as sodium chloride or magnesium sulfate, is added to the mixture. The addition of these salts reduces the solubility of polar compounds in the aqueous phase so that they transfer into the organic phase in a process known as “salting out”. This extraction technique offers multiple advantages, including the possibility of employing polar or moderately polar solvents, unlike in most of the LLE techniques. This is especially valuable for compounds that have higher affinities to polar solvents, broadening its applicability to include wider ranges of compounds. For the extraction of benzimidazole anthelmintic drugs from three types of milk (cow, sheep and goat), Tejada-Casado et al. applied an SALLE approach, obtaining recoveries that ranged from 79.1 to 99.6% [55].

In ALLME, a water-immiscible (organic) solvent is mixed with the aqueous sample that contains the target analytes. Similar to the mechanism of DLLME, air is injected through a fine needle. This produces fine bubbles in the sample solution, leading to the dispersion of the organic phase into microdroplets within the aqueous phase. ALLME also offers multiple advantages such as simplicity and improved efficiency due to the large surface area provided by the extraction microdroplets [82]. Mogaddam et al. applied ALLME for the extraction of aflatoxin M1 from milk samples before their analysis using HPLC-FLD with an extraction recovery of 87% [83].

### 4.8. QuEChERS

QuEChERS, which stands for “Quick, Easy, Cheap, Effective, Rugged, and Safe”, is another sample preparation method in which the sample is mixed with a solvent or a mixture of solvents (polar and nonpolar). Salts such as magnesium sulfate and sodium chloride are added to facilitate phase separation and concentrate analytes in either the polar or nonpolar layer. The extract is further purified using an extraction solid phase, combining aspects of SPE and LLE in a simplified form and on a smaller scale. The QuEChERS extraction approach offers multiple advantages, such as simplicity, selectivity, a reduction in treatment steps and subsequent shortening of extraction time, less solvent consumption, and cost-effectiveness [84].

As an example, Bang Ye et al. used this extraction procedure to extract nineteen quinolone antibiotics from goat’s milk samples prior to their analysis via UPLC–MS/MS [32]. They used 5% formic acid in acetonitrile as the extracting solvent; anhydrous sodium sulphate, NaCl, sodium citrate, and disodium hydrogen citrate as the extraction powder; and anhydrous sodium sulphate and C18 as the purification powder. This extraction process yielded recoveries in the range of 73.4–114.2% for the target antibiotics. The QuEChERS extraction method was chosen for the extraction of different classes of EPs from milk, including pesticides, EDCs, and pharmaceuticals [31,85,86].

### 4.9. MAE and UAE

Innovations in sample extraction and treatment techniques are continuous. Innovation not only occurs due to the development of new types of sorbents and extraction devices, but also via the integration different forms of energy, such as microwave and ultrasound, into extraction procedures.

Microwave-assisted extraction (MAE) is a nontraditional type of extraction in which microwave radiation is used to heat the sample matrix and the extraction solvent, which in turn enhances and accelerates the extraction process by allowing for solvent penetration into the matrix. Microwave-assisted solid-phase extraction offers many advantages, including reductions in the required volume of both the sample and harmful organic solvents, in addition to shorter extraction times due to the aid of the uniform heat effect, the automated nature of this technique, and its ability to simultaneously instead of sequentially extract multiple samples [87,88].

The fact that MAE often requires fewer volumes of organic solvents and shorter extraction times compared to traditional extraction techniques subsequently leads to less waste being generated and released into the environment, which makes this type of techniques more environmentally friendly [87,88]. On the other hand, there are some limitations that should be considered before choosing MAE as the technique of extraction, such as the tolerance of the sample to microwave radiation without being thermally degraded.

Although MAE is particularly well suited for solid samples, it has shown to be efficiently adopted for the extraction of analytes from liquid samples when combined with other types of extraction techniques, such as LLE and SPE [87]. Although the MAE approach was recently applied for the analysis of different pollutants in food matrices [89,90,91], it was not reported for the extraction of EPs from milk within the time period covered in this review.

Similar to MAE, in ultrasound-assisted extraction (UAE), ultrasound waves are used to generate localized heat in the sample, facilitating extraction procedures. Kubica et al. applied UA solvent extraction for the extraction of nineteen phenolic compounds from powdered milk and infant and toddler ready-to-feed milk, with recoveries ranging from 31% to 120%. This extraction approach was only seldom applied for the purpose of extraction of EPs from milk [36].

### 4.10. GDME

As time passes, advancements in extraction techniques continue. Among others, gas-diffusion microextraction (GDME) is a recent and innovative extraction technique in which the microextraction process is combined with gas diffusion. This assists in the adsorption of volatile and semi-volatile analytes to the microextraction fiber or syringe by creating a pressure difference that drives the target analytes from the liquid sample through the extraction device or membrane. Lobato et al. employed a GDME system for the extraction of a group of organochlorine pesticides from milk samples prior to their analysis (GC-ECD and GC-MS), achieving recoveries above 90% [92]. Although this extraction approach offers multiple advantages such as low solvent consumption, shorter analysis time, and high sensitivity, sample type has to be taken into account when thinking of this approach for use in extraction as GDME is well suited for volatile samples and may be not the optimal approach for the extraction of complex matrices that contain wide ranges of volatile compounds.

### 4.11. EME

One of the recent advanced forms of extraction is electromembrane extraction (EME). In EME, an electric field is applied to drive the migration of analytes through a supported liquid membrane (SLM), which is typically a porous membrane impregnated with an organic solvent that acts as an extraction phase. The sample solution containing the target analytes is placed on one side of the SLM and this is considered the donor solution. An electrolyte solution is placed on the other side as the receiving or acceptor solution. Under the effect of the electric field, the target charged analytes migrate from the sample solution towards the acceptor solution, passing through the SLM. Huang et al. provided the most recent review, explaining and covering the fundamental aspects of EME, advancements in device and operation modes, and possible applications [93].

In the context of EPs and milk, Aghaei et al. used EME for the extraction and preconcentration of ampicillin antibiotic residues in cow milk samples prior to their analysis using HPLC-UV [94]. The EME procedures involved the optimization of SLM composition, which were mainly composed of octan-1-ol, reduced graphene oxide, and silver nanoparticles. A high enrichment factor of 295 was obtained, corresponding to an extraction recovery of 37%.

## 5. Applications of Chromatographic Techniques for the Analysis of Different EP Categories in Milk

A massive body of literature has been devoted to the analysis of EPs in milk by using combinations of different extraction procedures and various subsequent chromatographic analytical techniques. Although different categories of EPs were analyzed in milk, the major emphasis of the selected research studies was on four categories: pharmaceuticals, endocrine-disrupting chemicals (EDCs), mycotoxins, and pesticides. The residues of other categories of EPs were also found in milk in a number of studies. These included hormones, food preservatives, adulterants, and per- and polyfluoroalkyl substances (PFASs).

### 5.1. Pharmaceuticals

Veterinary drugs and antibiotics are extensively used in veterinary medicine and livestock production because of their importance in treating and preventing various diseases, enhancing feed efficiency, and promoting growth rates [95,96]. They are commonly given to animals to treat prevalent cattle ailments such as mastitis, endometritis, bronchopathies, pneumonia, and lameness [15,19]. However, the misuse of these drugs or failure to adhere to the recommended withdrawal periods post-treatment can result in the accumulation of their residues in the animal’s body, animal’s food, and the environment [15,24]. The remaining residues in the animal’s body can contaminate food items like milk, egg, and meat [95]. Veterinary drug residues in milk not only directly impact human health, but also affect the quality of dairy products consumed by humans [15]. Health risks associated with drug residues in milk encompass allergic reactions, cellular mutations, teeth hypoplasia, bone marrow aplasia, and imbalances in the intestinal microbiome [17,97]. Moreover, these residues can induce reproductive system abnormalities, elevate cancer risks, impair the immune system, and cause disruptions in the endocrine and nervous systems [97]. Consequently, to protect human health and ensure food safety, states and international regulatory agencies such as the People’s Republic of China, the European Union (EU), and the Codex Alimentarius Commission (CAC) have established maximum residue limits (MRLs) for veterinary drug and antibiotic residues in milk. These limits act as precautionary benchmarks, aimed at guaranteeing consumer safety [17]. To further illustrate the scope and depth of this concern, researchers have studied the presence of drug residues in dairy milk. Table 1 provides an overview of the most pertinent publications, from 2018 until present, on the detection and determination of veterinary drug and antibiotic residues in dairy milk that used LC and GC methods, coupled with different detection techniques. As reported in the literature, different groups of antibiotics have been used in veterinary medicine and livestock industry and large numbers of research studies have been devoted for their analysis using chromatography-based methods. The majority of selected research papers summarized in Table 1 focused on the determination of the tetracycline (TC) family of antibiotics. Owing to the antibiotic activity they exhibit against wide range of bacteria and microorganisms, TCs are excessively used as veterinary drugs [98,99]. Nonetheless, the improper handling of TCs can lead to the presence of their residues in animal-based food products, creating a substantial risk to consumers. Such risks encompass allergic reactions in susceptible individuals, chronic toxicity, and the development of antimicrobial resistance [98,99,100].

Other classes of antibiotics that were observed to be of major concern, include quinolones, were reflected in the number of studies depicting them in milk (Qs). Qs are non-steroidal synthetic antibiotics. Their affordability, low toxicity, and broad antibacterial activity have made them amongst the most used antibiotics in the livestock industry for the treatment of some diseases, including respiratory diseases associated with the two bacterium species Mannheimia haemolytica and Pasteurella multocida [138,139]. However, their excessive use and the subsequent presence of residues in food of animal origins like milk can pose substantial safety and health concerns owing to their carcinogenicity and antibiotic resistance [112,140]. Other classes of antibiotics, including beta-lactams (β-lactams), macrolides, sulfonamides (SAs), glycopeptides, and amphenicol antibiotics, were also reported in relatively fewer studies within the time period covered in this review [106,119,121,141].

In addition to antibiotics, a number of studies have developed chromatography-based analytical methods for the determination of residues of other types of pharmaceuticals and veterinary drugs in milk such as anthelmintics, diuretics, and NASIDs [125,130,131].

Although a variety of analytical methods was employed for the determination of these classes of pharmaceuticals in milk, the combination of liquid chromatography and tandem mass spectrometry was the method of choice in the majority of studies, as can be observed from Table 1’s data.

### 5.2. Endocrine-Disrupting Compounds

Food packaging serves a crucial function in the food sector; it extends shelf life and protects food contents from biological and chemical alterations post-processing [56]. Packaging materials comprise various components, including polymers, plasticizer additives, and endocrine-disrupting compounds (EDCs) [142]. EDCs are exogenous substances that can interfere with the endocrine system, either by inhibiting the primary hormone functions or mimicking their actions [1,143]. A primary concern with EDCs is their migration from the packaging or storage materials into the food [143]. Another route for EDCs to enter the food chain is via contaminated animal feed [144]. Toxic EDCs, such as phthalates and bisphenols, have the potential to bioaccumalate, posing threats to human health [142]. They are associated with various physiological disruptions and are linked to diseases like diabetes, obesity, reproductive disorders, cardiovascular disease, congenital disabilities, and breast cancer [143]. Both phthalates and bisphenols can enter the human body through dermal absorption from consumer products or via ingestion due to migration from the packaging material to food [142,145]. It is worth noting that these migration rates can increase at high temperatures [142].

Bisphenols and phthalates have a lipophilic nature. If animal feed becomes contaminated with these chemicals, they can accumulate in the livestock’s adipose tissue and may subsequently be excreted into the milk [33]. Given milk’s crucial role in children’s nutrition, special attention should be given to it. Milk is often consumed in plastic bottles; thus, it is assumed that bisphenols and phthalates can easily migrate from packaging materials into the milk due to the lipophilic nature of both the chemicals and the milk itself [144].

In addition to phthalates and bisphenols, concerns regarding endocrine-disrupting effects have been raised for other chemical substances, such as parabens. Parabens, including methyl paraben, ethyl paraben, propyl paraben, and butyl paraben, are esters of para-hydroxybenzoic acid. Parabens serve as preservatives of antimicrobial activity and high stability in a broad array of cosmetics, personal care products, food products, and pharmaceuticals [146,147,148]. Exposure to high levels of parabens induces alterations in normal hormonal levels, negatively impacting reproductive system, thyroid functions, and dermal system among others. Similar to other types of EDCs, parabens can find their way into milk through different sources, including contaminated feed, food packaging, and the contaminated surrounding environment.

Therefore, determining the levels of EDCs present in dairy milk is essential for consumer safety. Table 2 provides a summary of the most relevant publications on the determination of EDCs in dairy milk using LC- and GC-based analytical techniques.

### 5.3. Pesticides

Pesticides play a pivotal role in agriculture. They are not only used to boost yield and ensure the quality of crops, but also to control diseases and deter pests [124]. These chemicals can be applied to the feed and fodder of livestock. Additionally, they can be applied directly to breeding animals or their habitats to protect against pests and pathogens or to treat diseases caused by them [31]. However, these chemicals do not solely affect their intended targets. Residues can make their way to non-targeted species, including livestock. Due to the persistent nature of pesticides, their residues may accumulate in animal tissues and subsequently find their way into the human food chain [31,161].

Owing to milk’s fat-rich content, it is particularly susceptible to contamination by pesticide residues due to their lipophilic nature [143,162]. Milk’s nutritional benefits make it a primary dietary component, especially for children and infants [37]. While milk is a rich source of nutrients, its contamination with pesticide residues can have detrimental effects on consumer health. Consuming milk contaminated with these residues can lead to immediate health concerns, such as lacrimation, seizures, headaches, and abdominal pain [162]. In the long term, exposure to these toxic chemicals can raise the risk of severe health problems, including genetic disorders, nervous system complications, cancer, and congenital disabilities [37]. In response to these risks, international regulatory authorities have set MRLs for pesticide residues in milk in order to ensure public health. Numerous studies have been conducted to investigate and quantify the levels of pesticide residues in dairy milk by employing chromatographic techniques [31,81,92,163]. Table 3 offers a comprehensive summary of these key publications published in the time period of 2018–2023.

### 5.4. Mycotoxins

Mycotoxins are secondary metabolites produced by specific types of fungi belonging mainly to the Aspergillus, Penicillium, and Fusarium genera that infest and colonize many crops in fields during storage and during processing and preparation [178,179].

When food-producing animals consume contaminated feed, mycotoxins undergo metabolism and biotransformation, ultimately being transferred to eggs, milk and meat. They pose potential health risks owing to their hepatotoxic, carcinogenic, and genotoxic effects [180,181]. Among different types of mycotoxins such as zearalenone, ochratoxins, sterigmatocystin, and fumonisins, aflatoxins have gained popularity and received special attention [182,183]. Aflatoxins (AFs), which are mainly produced by Aspergillus flavus, Aspergillus nominus, and Aspergillus parasiticus fungi, are among the most studied types of mycotoxins in the literature. This is due to their acutely toxic properties, in addition to their carcinogenicity, teratogenicity, mutagenicity, and hepatotoxicity [180,183,184,185].

Aflatoxin B1 (AFB1) is the most prevalent form of aflatoxins that contaminate crops. AFB1 is known to be highly toxic, and it is classified as a human carcinogen (group 1) by the International Agency for Research on Cancer (IARC) [186,187].

When milk-producing animals are fed with AFB1-contaminated feed, milk undergoes a hydroxylation process via the action of the cytochrome P450 enzyme producing the hydroxylated metabolite AFM1, which also demonstrates toxic effects on humans [188,189]. Several regulatory organizations have set maximum residue limits (MRLs) for AFM1 and other mycotoxins in milk and food products. Flores-Flores et al. summarized some of those regulations [190,191].

As milk is a very popular and a widely consumed nutritious meal, numerous research studies have been devoted to analyzing and determining the aflatoxins and other types of mycotoxins present in milk using chromatography-based analytical techniques. Table 4 provides an overview of those methods and their analytical performance parameters.

### 5.5. Other Emerging Pollutants

Considerable attention has been dedicated to drugs, EDCs, mycotoxins, pesticides, and their residual levels in milk. However, in this review, we expand our discussion to encompass other types of contaminants, including hormones, per- and polyfluoroalkyl substances (PFASs), polyaromatic hydrocarbons (PAHs), polychlorinated biphenyls (PCBs), melamine (as a non-protein nitrogen supplement), and formaldehyde.

The presence of hormones in edible matrices, such as milk, has raised concerns due to their significant impact on the endocrine system and cell signaling, leading to disruptions in the homeostasis of those who consume them [27]. Moreover, elevated levels of estrogen have been associated with breast, uterine, and ovarian cancers in women [205]. Natural and synthetic steroid hormones are extensively employed in cattle to treat certain diseases, promote growth, and address reproductive disorders [206]. However, exceeding acceptable dosages, improper injection, or the use of banned hormones can result in the presence of their residues in milk. Therefore, it is imperative to investigate the extent of hormonal contamination in milk to ensure food safety.

PFASs are highly stable compounds, leading to their extensive use in food packaging materials and flame retardants. However, their resistance to biodegradation results in their accumulation in the environment. Milk is considered one of the most contaminated food items, with various PFASs [42]. The entry of PFASs into milk and dairy products can occur through processing and packaging or via contaminated animal feed. PFASs can pose serious threats to human health, including cancer, allergies, and infertility [42]. Hence, the determination of PFASs in milk and food matrices has garnered significant attention from researchers. However, a comprehensive knowledge and understanding regarding their occurrence, migration, associated risks, and tolerable limits is still required.

Melamine, a nitrogen-rich organic compound, finds applications in different industries including plastics, adhesives, coatings, amino resins, and laminates [207,208]. Beyond its typical commercial and industrial uses, melamine, as a cheap and available substance rich with nitrogen, is illegally introduced into milk and dairy products to artificially and falsely boost their apparent protein content. Various health effects have been reported as being induced by melamine, including nephrolithiasis, stones formation, bladder carcinoma, and kidney inflammations [209,210]. In 2008, China experienced several human death cases arising from kidney failure, in addition to other health complications, as a result of melamine adulteration [211]. To protect public health, intensive guidelines and regulations were introduced by several organizations and authorities to control the use of and exposure to melamine [212]. Therefore, analytical methods are being continuously developed for melamine tracking in milk and milk products [48,49,213].

Various chemical substances are being added to foods under the category of food adulterants and preservatives in order to elongate and extend their shelf life. Such chemicals include formaldehyde (FA), which is the most common and the most accessible. FA can reach food matrices including milk via several pathways, including its direct application as a preservative, and its migration from the packaging material or from the contaminated environment. Consequently, such contamination can lead to severe health impacts owing to the toxic and carcinogenic nature of FA [214,215]. Therefore, there is a need for to pay special interest to monitoring efforts to track the presence of FA in food matrices, particularly in highly consumed products like milk.

Due to their physical and chemical stability, polychlorinated biphenyls (PCBs) are widely used in different industrial applications such as paints, rubber, and plastics industries [34]. However, due to their tendency to bioaccumulate in adipose tissue, they can be transferred to food of animal origin such as milk. PCBs are toxic chemicals that can lead to cancer and neurological, reproductive, and immune system disorders [216]. Consequently, their monitoring in milk is of a significant importance. Similarly, polyaromatic hydrocarbons (PAHs), a type of organic pollutants that can contaminate food via different ways such as environmental contamination or during food processing and preparation, are a matter of concern for public health due to their mutagenicity, carcinogenicity, and immune system suppression effect [217,218,219]. This underscores the need for developing analytical methods for tracking and quantifying such pollutants in milk.

Table 5 provides data from previous studies regarding the presence of hormones, PFASs, PCBs, PAHs, melamine, and other contaminants in dairy milk, in addition to multiclass residues that are simultaneously analyzed using the same analytical methods.

## 6. Concluding Remarks and Future Directions

Chromatography-based analysis techniques are continuously evolving in order to precisely determine the presence of EPs in milk. Commonly analyzed residues include veterinary drugs, especially antibiotics; EDCs, such as phthalates and bisphenols; pesticides; and mycotoxins. Several studies have also explored other categories of EPs, encompassing hormones, food adulterants, PCBs, and PFASs.

Due to its range of detector options, chromatography facilitates the application of various analytical methods tailored for selectively and sensitively determining different categories of EPs. Although LC and GC coupled to MS remain the most prevalent combinations, other reported techniques include LC-UV, LC-FLD, and GC-FID. Among the 155 studies included in this review, LC paired with MS emerged as the most frequently employed method for determining EPs in milk, accounting for more than 45% of all reported techniques.

In the analysis of veterinary drug residues, LC-MS/MS emerged as the most prominent method, followed by the use of LC combined with UV. Notably, LC coupled to FLD was reported in only one study analyzing the residues of veterinary drugs. Interestingly, no studies within the reviewed period utilized GC-MS to analyze veterinary drug residues in milk, suggesting an unexplored avenue for future research. On the other hand, in the examination of EDC residues, including phthalates, bisphenols and parabens, the most commonly employed analytical techniques were LC coupled to UV and FLD, surpassing both LC-MS and GC-MS. However, regarding pesticide residues, both LC- and GC-based techniques were used in comparable numbers of studies. Finally, in the determination of mycotoxins residues, LC coupled to FLD was the dominant method of choice for performing analysis.

Although the aforementioned chromatographic techniques, especially LC-MS and GC-MS, were heavily utilized and proved to be well suited for analyzing the majority of EPs in food matrices like milk, there is a suggestion of the need to explore other types of chromatographic techniques. These may include capillary liquid chromatography (CLC), micellar liquid chromatography (MLC), supercritical fluid chromatography (SFC), ion chromatography (IC), and capillary electrophoresis (CE). Moreover, advancements in chromatographic instrumentation and column technologies could further enhance the performance and efficiency of chromatography-based methods for analyzing EPs in complex food samples like milk. These innovations encompass the integration of high-resolution mass spectrometry (HRMS), monolithic chromatographic columns, multidimensional chromatography, portable miniaturized LC systems, and microfluidic devices.

Milk, which is a complex matrix due to its content of fat, proteins, and vitamins, requires a pretreatment step for purification and preconcentration. Various approaches have been developed and improved from the classical SPE and LLE techniques to ensure the specific and efficient extraction of different EPs from milk samples before their assessment using chromatographic techniques.

Among all the reviewed papers, SPE and its variations were the most commonly applied extraction approaches, constituting approximately 45% of the total studies. Specifically, SPE and its different modes were the predominant approaches used to extract residues of both veterinary drugs and EDCs, while QuEChERS-based extraction was the most frequently applied method for pesticide residues. A diverse array of materials was reported to be used as SPE adsorbents, including traditional silica NPs, C8, C18, urea, MOFs, COFs, and MIPs. MOFs, COFs, MIPs, and carbon nanomaterials, reported as solid-phase adsorbents in several studies covered in this review, are anticipated to undergo further development and enjoy widespread utilization for the purpose of EP separation. These materials are expected to gain more attention due to their promising advantages, such as high surface areas, tailorable properties and structures, and exceptional chemical stability.

Regarding future research and the growing emphasis on green chemistry, it is noteworthy that biosorbents like cellulose, lignin, and chitin hold promise as candidates for exploration and incorporation as novel green adsorbents in the extraction of various EPs from milk. Their abundance and environmentally friendly nature contribute significantly to the overall greenness of the analytical method.

While the sample preparation process is crucial, particularly in complex matrices like milk, it inevitably adds time to the overall duration of analysis. This temporal factor, especially during large-scale and routine analyses, can be considered a drawback. Consequently, trends towards automated extraction are expected to accelerate in the future, driving the increased utilization of online and in-line extraction methods.

According to the Food and Agriculture Organization (FAO), cows contribute approximately 82% of the world’s milk production, followed by buffaloes at 13%, goats at 2%, sheep at 1%, and camels at 0.4%. Consequently, the majority of the reviewed research studies focused on the analysis of EPs in cow milk, representing more than 80% of the studies. International organizations and states such as the People’s Republic of China, the EU, and the CAC have established MRLs for various EPs in cow’s milk. However, the MRLs of these compounds might not be available in other types of milk due to the limited research investigating and monitoring EPs in other types of milk. Therefore, it is imperative to develop analytical methods specifically tailored for the analysis of EPs in these diverse milk types. Due to significant variations in fats, vitamins, and protein composition among different milk types, distinct extraction procedures should be further developed and validated before conducting chromatographic analysis. Camel milk, in particular, is one of the primary dietary components in many parts of the world, including Gulf countries, and the Middle East. Its increasing popularity is attributed to its unique nutritional values and reported therapeutic properties in numerous studies. Its distinct composition makes it a valuable yet challenging subject for study. Addressing these knowledge gaps in research data will not only enhance our understanding of this topic but also aid regulatory agencies in making informed decisions and establishing suitable MRLs.

Despite the extensive body of research dedicated to the analysis of various categories of EPs in milk, there are still unexplored areas in this field. Other categories of EPs remain understudied, such as personal care products, dioxins, volatile organic compounds (VOCs), flame retardants, hormones, nitrates, and nitrites. Knowledge gaps persist regarding the presence of EPs, their contamination levels in pathways in milk, and their potential impacts.

## Figures and Tables

**Table 1:** Overview of the performance of analytical methods for the extraction and determination of pharmaceuticals residues in dairy milk.

Target EPs	Category	Extraction Method	Analysis Technique	Matrix	Analytical Parameters	Conc. in Real Samples	Country	Ref.
Tetracycline (TC), oxytetracycline (OTC), chlortetracycline (CTC), doxycycline (DC)	TC antibiotics	FPSE	HPLC-UV	Milk	LOD: 15 μg/kg	ND	Greece	[101]
					LOQ: 50 μg/kg			
					CCα: 103.2–108.1 μg/kg			
					CCβ: 108.6–114.3 μg/kg			
					R: 88.9–122.4%			
					RSD: ≤14.5%			
TC, OTC, CTC	TC antibiotics	MSPD	UHPLC–MS/MS	Milk powder	LOD: 0.217–0.318 ng/g	ND	China	[102]
					LOQ: 0.723–1.060 ng/g			
					LR: 1–100 ng/g R^2^: 0.998–0.999			
					R: 84.7–93.9%			
					RSD: <7.5%			
TC, OTC, DC	TC antibiotics	MSPE-DLLME	HPLC-UV	Bovine milk	LOD: 1.8–2.9 μg/L	Spiked	Iran	[51]
					LOQ: 6.1–9.7 μg/L			
					LR: 10.0–200.0 μg/L			
					R^2^: >0.9929			
					RSD: 2.5–8.8%			
					R: 70.6–121.5%			
OTC, CTC, TC	TC antibiotics	MSPE	HPLC-UV	Milk	LOD: 1.29–2.31 ng/mL	ND	China	[103]
					LOQ: 4.26–7.62 ng/mL			
					LR: 5–250 ng/mL			
					R: 79–109%			
					RSD: <7.25%			
TC, OTC, CTC, DC	TC antibiotics	MSPE	HPLC-UV	Milk	LOD: 1.03–1.31 μg/L	ND	China	[100]
					LOQ: 3.46–4.41 μg/L			
					LR: 5.0–700 μg/L R^2^: 0.9991–0.9996			
					R: 86.7–98.6%			
					RSD: 1.4–5.7%			
OTC, TC, CTC, DC	TC antibiotics	QuEChERS	HPLC-DAD	Milk	LOD: 15 μg/kg	ND	Greece	[104]
					LOQ: 50 μg/kg			
					CCα: 100.3–105.6 μg/kg			
					CCβ: 100.6–109.7 μg/kg			
					R: 83.07–106.3%			
					RSD: <15.5%			
Sulfadiazine (SD), sulfapyridine (SP), sulfathiazole (SZ), sulfamethazine (SMZ), sulfamethoxypyridazine (SMP), sulfachloropyridazine (SCP), sulfamethoxazole (SMX), sulfisoxazole (SIX), sulfadimethoxine (SDM), sulfaquinoxaline (SQX)	SA antibiotics	SPME	HPLC-DAD	Milk	LOD: 0.077–0.350 μg/L	NS	Greece	[105]
					LOQ: 0.23–1.05 μg/L			
					LR: 0.5–150 μg/L R^2^: >0.9964			
					R: 88–97%			
					RSD: <10%			
					CCα: 111.2–113.6 μg/L			
					CCβ: 122.6–127.4 μg/L			
Sulfanilamide (SN), SD, SMZ, sulfamerazine (SM), SP, SZ, SMP, SMX, SDM	SA antibiotics	SPE	HPLC-UV	Milk	LOD: 3.0–12.3 μg/kg	ND	China	[106]
					LOQ: 10–43 μg/kg			
					LR: 20–1000 μg/kg			
					R: 80.7–101.3%			
					RSD: <8.5%			
SN, SD, SZ, and sulfamethizole (SMT)	SA antibiotics	CPME	HPLC-DAD	Milk	LOD: 16.7 μg/kg	ND	Greece	[107]
					LOQ: 50 μg/kg			
					LR: 50–2000 μg/L			
					CCα: 104.5–111.4 μg/kg			
					CCβ: 109.4–118.1 μg/kg			
					Absolute R: 12.1–18.1%			
					RSD: <11.2%			
SZ, SME, SDM, Sulfamonomethoxine (SMM)	SA antibiotics	d-MSPE	HPLC-DAD	Milk	LOD: 2.5, 5.0 μg/kg	SME: 15.1 μg/kg	Thailand	[108]
					LOQ: 7.5–10.0 μg/kg			
					LR: 2.5–150.0 μg/kg R^2^: >0.997			
					R: 83.0–99.2%			
					RSD: <6%			
Ciprofloxacin (CIP), fleroxacin (FLE), and oxolinic acid (OXO), danofloxacin (DAN), difloxacin (DIF), flumequine (FLU), lomefloxacin (LOM) marbofloxacin (MAR), nalidixic acid (NAL), norfloxacin (NOR), pefloxacin (PEF), pipemidic acid (PIP), sarafloxacin (SAR), enrofloxacin (ENR), levofloxacin (LEV), trovafloxacin (TRFX), orbifloxacin (ORB), ofloxacin (OFl), and cinoxacin (CIN)	Q antibiotics	QuEChERS	UPLC–MS/MS	Goat’s milk	LOQ: 5 ppb	ND	Taiwan	[32]
					R^2^: >0.9853			
					R: 73.4–114.2%			
					CV: <15%			
DIF, ORB, Sparfloxacin (SPA), SAR, FLE, MAR, OFL, ENR, DAN, LOM, PEF, CIP, ENO, NOR, PIP, CIN, OXO, NAL	Q antibiotics	MSPE	HPLC-MS/MS	Milk	LOD: 3.1–13.3 ng/L	CIP (2 μg/L), DAN (0.66 μg/L), (One sample)	China	[109]
					LOQ: 10.4–44.2 ng/L			
					LR: 0.05–10 μg/L R^2^: 0.9975–0.9996			
					R: 82.4–103.9%			
					RSD: 2.9–15.1%			
OFL, NOR, CIP, ENR, DIF, PEF, DAN	Q antibiotics	MSPE	HPLC–MS/MS	Milk	LOD: 0.35–1.5 μg/L	ND	China	[110]
					LOQ: 1.2–4 μg/L			
					LR: 1.5–200 μg/L R^2^: >0.99			
					R: 75–88.3%			
					RSD: 5.3–9.1%			
CIN, CIP, DAN, DIF, enoxacin (ENO), ENR, FLU LOM, MAR, moxifloxacin (MOX), NAL, NOR, OFL, OXO, PIP, piromidic acid (PIRO), SAR	Q antibiotics	SBSE	UHPLC–MS/MS	Raw cow milk	LOD: 0.1–1.0 μg/kg	CIP, ENR and MAR 2.7–35.3 μg/kg	Spain	[111]
					LOQ: 0.5–4.0 μg/kg			
					LR: 0.5–150 μg/kg R^2^: 0.99–0.999			
					R: 88.0–114.0%			
					RSD: 2.0–14.0%			
					CCα: 30.7–106.1 μg/kg			
					CCβ: 31.3–122.0 μg/kg			
OFL, NOR, CIP	Q antibiotics	SPE	HPLC-FLD	Cow milk	LOD: 39, 30, 33 ng/L	ND	Spain	[112]
					LOQ: 120, 92, 100 ng/L			
					LR: 1.8–250 μg/L			
					R: 60–70%			
					RSD: 4–13%			
CIP, ENR, NOR, LOM, ENO, SPA	Q antibiotics	SPE	HPLC-UV	Milk	LOD: 2.8–5.1 ng/g	ND	China	[47]
					LOQ: 9.5–17 ng/g			
					LR: 10–2000 ng/g R^2^: 0.9972–0.9997			
					R: 85.8–117.9%			
					RSD: ≤9.4%			
					CCα: 102.1–105.1 ng/g			
					CCβ: 108.3–116.0 ng/g			
CIP, ENR, LOM, PEF, LEV gatifloxacin (GAT)	Q antibiotics	MSPE	HPLC-DAD	Milk	LOD: 0.25–0.5 ng/g	ND	China	[113]
					LR: 2.5–1500 ng/g R^2^: >0.9996			
					R: 81.05–98.75			
					RSD: 1.5–4.3%			
PEF, CIP, ENR, LOM, SAR	Q antibiotics	MSPE	HPLC-MS/MS	Milk	LOD: 0.04–0.10 ng/g	Spiked	China	[114]
					LOQ: 0.1–0.2 ng/g			
					LR: 0.1–200 ng/g r: 0.9991–0.9997			
					R: 78.1–95.2%			
					RSD: 1.2–7.9%			
ENO, FLE, OFL, NOR, PEF, LOM	Q antibiotics	MSPE	HPLC-UV	Milk	LOD: 0.05–0.20 μg/L	ND	China	[71]
					LOQ: 0.19–0.71 μg/L			
					LR: 0.5–200 μg/L *r*: 0.9982–0.9996			
					R: 90.4–101.2%			
					RSD: 3.5–4.7%			
Ampicillin, benzylpenicillin, amoxicillin, oxacillin, and cloxacillin	β-lactam antibiotics	D-m-SPE	UPLC–MS/MS	Cow, goat, and sheep milk	LOD: 0.03–0.20 μg/kg	ND	Iran	[115]
					LOQ: 0.17–0.68 μg/kg			
					LR: 0.1–300 μg/kg R^2^: 0.9978–0.9995			
					R: 87–107%			
					RSD: ≤5.8%			
					CCα: 4.1–31.0 μg/kg			
					CCβ: 4.3–32.1 μg/kg			
Ampicillin	β-lactam antibiotics	EME	HPLC-UV	Cow milk	LOD: 0.6 μg/L	ND	Iran	[94]
					LR: 2–100 μg/L			
					R^2^: 0.995			
					R: 37–45%			
					RSD: <7.1%			
Thirty-two antibiotics	β-lactam antibiotics	d-SPE	UHPLC-MS/MS	Bovine milk	LOD: 0.0090–1.5 μg/kg	NS	Ireland	[116]
					LOQ: 0.030–5.0 μg/kg			
					R^2^ ≥ 0.98			
					R: 91–130%			
					RSD: 1.4–38.6%			
					CCα: 2.1–133 μg/kg			
					CCβ: 2.4–182 μg/kg			
Ceftiofur	β-lactam antibiotics	Online SPE	HPLC-MS/MS	Bovine milk	LOD: 0.1 μg /L	ND	Brazil	[117]
					LOQ: 0.7 μg /L			
					R^2^: >0.98			
					R: 73.4–111.3%			
					RSD: <15%			
Thirty-one compounds	Macrolide antibiotics	QuEChERS	UPLC–MS/MS	Milk	LOD: 0.1–0.5 μg/L	LOD < C < LOQ	China	[118]
					LOQ: 0.5–2.0 μg/L			
					LR: 1–200 μg/L R^2^: >0.990			
					R: 81.07–110.1%			
					RSD: <5.1%			
Azithromycin (AZI), clarithromycin (CLA), erythromycin (ERY), lincomycin (LIN), roxithromycin (ROX)	Macrolide antibiotics	mini-SPE	UHPLC-Q-TOF/MS	Bovine milk	LOD: 0.017–0.76 μg/kg	LIN: 2.16 μg/kg AZI: 174.94 μg/kg ERY: 7.91 μg/kg CLA: 24.04 μg/kg ROX: 13.87 μg/kg	China	[119]
					LOQ: 0.054–2.52 μg/kg			
					MDL: 0.027–1.01 μg/kg			
					MQL: 0.026–0.96 μg/kg			
					R^2^: >0.99			
					R: 77.91–105.34%			
Gamithromycin	Semisynthetic macrolide antibiotics	SPE	UHPLC-MS/MS	Milk	LOD: 0.30–0.40 μg/kg	ND	China	[120]
					LOQ: 0.80–1.0 μg/kg			
					LR: 1.0–200 μg/kg R^2^: >0.99			
					R: 109.8–114.8%			
					RSD: 1.4–6.8%			
Lincomycin (LIN)	Lincosamide antibiotics	CSMISPE	HPLC-UV	Pasteurized milk	LOD: 0.02 μg/mL	0.10–0.61 μg/mL	Iran	[66]
					LOQ: 0.08 μg/mL			
					LR: 0.08–2 μg/mL R^2^: 0.999			
					R: 80–89%			
					RSD: ≤4.03%			
Vancomycin, teicoplanin, telavancin, oritavancin, dalbavancin	Glycopeptide antibiotics	SPE	UHPLC–MS/MS	Milk	LOD: 0.33 μg/kg	Spiked	China	[121]
					LOQ: 1.00 μg/kg			
					R^2^: 0.9987–0.9999			
					R: 83–102%			
					RSD: 1–6.8%			
Vancomycin and norvancomycin	Glycopeptide antibiotics	Online SPE	LC-HRMS	Milk	LOD: 0.15 μg/kg	Spiked	China	[44]
					LOQ: 0.5 μg/kg			
					LR: 0–200 ng/mL R^2^: >0.9983			
					R: 80.00–92.96%, 80.68–91.31%			
					RSD: 4.90–9.35%			
Vancomycin and norvancomycin	Glycopeptide antibiotics	SMISPE	LC–MS/MS	Milk	LOD: 0.5 μg/kg	ND	China	[122]
					LOQ: 1.0 μg/kg			
					LR: 0.5–50 μg/kg			
					R: 83.3–92.1%			
					RSD: <16.8%			
Chloramphenicol (CAP)	Amphenicol antibiotics	MSPE	HPLC-UV	Milk	LOD: 0.24 μg/L	ND	China	[123]
					LOQ: 0.79 μg/L			
					LR: 7–1.0 × 10^3^ μg/L R^2^: 0.9994			
					R: 80.5–105.0%			
					RSD: 5.3–8.9%			
Chloramphenicol (CAP)	Amphenicol antibiotics	SS-DMNF-ME	HPLC-UV	Milk	LOD: 0.22–0.25 ng/mL	ND	Iran	[124]
					LOQ: 0.73–0.85 ng/mL			
					LR: 0.9–250 ng/mL R^2^: ≥0.982			
					R: 91.4–95.1%			
					RSD: ≤4.16			
Closantel, nitroxynil, niclosamide, rafoxanide, eprinomectin, emamectin, levamisole, cymiazole, praziquantel, tetramisole, thiophanate, morantel, pyrantel, fluazuron, guaifenesin, carbendazim, cambendazole, trichlorfon	Anthelmintics	LLE	LC-MS/MS	Milk	LOD: 0.1–5 μg/kg	ND	Korea	[125]
					LOQ: 0.4–10 μg/kg			
					R^2^: ≥0.9752			
					R: 64.6–112.6%			
					RSD: ≤13.4			
Albendazole (ABZ), albendazole sulfoxide (ABZ-SO), benomyl (BEN), carbendazim (CBZ), fenbendazole (FBZ), fenbendazole sulfone (FBZ-SO_2_), fenbendazole sulfoxide (FBZ-SO), mebendazole (MBZ), mebendazole-amine (MBZ-NH_2_), thiabendazole (TBZ), 5-hydroxy-thiabendazole (5-OH-TBZ), triclabenda-zole (TCB), triclabendazole sulfone (TCB-SO_2_), triclabendazole sulfoxide (TCB-SO), Albendazole-2-aminosulfone (ABZ-NH_2_-SO_2_)	Anthelmintics	SALLE	CLC-UV	Cow, sheep and goat milk	LOD: 1.0–2.8 μg/kg	ND	Spain	[55]
					LOQ: 3.2–9.5 μg/kg			
					LR: 3.2–200 μg/kg R^2^: >0.9985			
					R: 79.1–99.6%			
					RSD: 1.6–14.2%			
Mebendazole	Anthelmintics	BSASLE + BUASLE	MLC-DAD	Milk	LOD: 0.2 ppm	1–7.4 ppm	India	[54]
					LOQ: 0.6 ppm			
					r^2^ = 0.9996			
					R: 98.5–99.8%			
					RSD: <5%			
Salicylic acid (SA), oxaprozin (OXP), diclofenac (DCF) and ibuprofen (IBF)	NSAIDs	UA-HDES-DLLME	HPLC-UV	Milk	LOD: 0.5–1 μg/L	ND	China	[126]
					LOQ: 1–5 μg/L			
					LR: 5–2000 μg/L R^2^: 0.994–0.999			
					R: 65.88–110.80%			
					RSD: 1.11–16.9%			
Ketoprofen (Ket), flurbiprofen (Flu), ibuprofen (Ibu), naproxen (Nap), and diclofenac sodium (DS)	NSAIDs	BSE	UPLC-DAD	Milk	LOD: 1.14–4.50 ng/mL	ND	China	[127]
					LOQ: 3.76–14.85 ng/mL			
					LR: 10–1000 ng/mL R^2^: 0.9988–0.9998			
					R: 80.8% to 110.2%			
					RSD: 2.3–3.5%			
Diclofenac sodium (DS)	NSAIDs	MSPE	HPLC-MS/UV	Milk	LOD: 10 ng/kg	28–68 ng/kg	China	[128]
					LOQ: 25 ng/kg			
					LR: 50–2000 ng/kg R^2^: 0.9996			
					R: 87–103%			
					RSD: 2.4–11.3%			
Spironolactone (SPRL), canrenone (CR), chlorothiazide (CTZ), hydrochlorothiazide (HCTZ), acetazolamide (AZ), furosemide (FSM), 4-amino-6-chlorobenzene-1,3-disulfonamide (ACB)	Diuretics	modified QuEChERS	HPLC–MS/MS	Milk	LOQ: 0.5–1.0 μg/kg	ND	China [129]	[130]
					R^2^: 0.9954–0.9999			
					R: 73–113.9%			
					RSD: 2.45–10%			
Chloramphenicol (CAP) Tetracycline (TC)	Multiclass antibiotics	MSPE	HPLC-DAD	Milk	LOD: 3.02, 3.52 ng/mL	CAP: (one sample): 53.3 ng/mL TC: (one sample): 75.8 ng/mL	Turkey	[52]
					LOQ: 9.63, 9.83 ng/mL			
					LR: 10.0–600.0 ng/mL R^2^: 0.9954, 0.9973			
					R: 94.6–105.4%			
					RSD: <4.0%			
SMM, OTC, CEF, MAR	Multiclass antibiotics	SPE	HPLC-DAD	Milk	LOD: 0.02 μg/mL	NS	Italy	[129]
					LOQ: 0.02 μg/mL			
					LR: 0.02–2.00 μg/mL R^2^: 0.993–0.998			
					R: 61.4–99.3%			
Sixty-two analytes	Multiclass antibiotics	SPE	UPLC-quadrupole/electrostatic field Orbitrap-HRMS	Goat milk	LOD: 0.5–1.0 μg/kg	Metronidazole: 2.45 & 5.02 μg/kg Enrofloxacin: 112.4 μg/kg	China	[131]
					LOQ: 5.0–10.0 μg/kg			
					LR: 0.5–100 μg/L R^2^: 0.9901–0.9998			
					R: 60.1–110.0%			
					RSD: <15%			
DC, TC, OTC, PNG, CAP, CIP, ENR	Multiclass antibiotics	MIL-based AALLME	HPLC–DAD	Milk	LOD: 0.09–0.21 ng/mL	TC:56–112 ng/mL OTC: 89–149 ng/mL CAP: 41 ng/mL (one sample)	Iran	[132]
					LOQ: 0.29–0.71 ng/mL			
					LR: 0.71–500 ng/mL R^2^: ≥0.994			
					R: 79–91%			
					RSD: 3.6–5.2%			
Twenty-two compounds	Multiclass antibiotics	MSPE	UPLC-MS/MS	Bovine milk	LOD: 0.04–0.19 μg/kg	0.54–97.18 μg/kg	Iran	[40]
					LOQ: 0.13–0.64 μg/kg			
					LR: 0.2–800 μg/kg			
					R^2^: 0.9958–0.9992			
					R: 85.9–107.5%			
					RSD: <9.2%			
					CCα: 0.10–111.3 μg/kg			
					CCβ: 0.13–125.8 μg/kg			
One hundred and three analytes	Veterinary drugs	Modified QuEChERS	UPLC-MS/MS	Cow milk and milk powder	LOD: 0.1–25 μg/kg	LIN: 10.2 ± 1.5 μg/kg (one sample)	China	[133]
					LOQ: 0.5–50 μg/kg			
					R^2^: 0.9902–0.9998			
					R: 31.1–120.7%			
					RSD: 2.34 to 19.2%			
Twenty-five analytes	Multiclass veterinary drugs	LLE	UHPLC–MS/MS	Commercial milk samples	LOQ: 0.1–4 ng/g	Clorprenaline: 0.5 ng/g and 0.47 ng/g hydrocortisone 0.78 ng/g (one sample)	China	[134]
					CCα: 0.008–113.68 ng/g			
					CCβ: 0.01–125.75 ng/g			
					LR: 0.1–384 ng/mL R^2^: 0.9901–0.9990			
					R: 65.9–123.5%			
					RSD: ≤11.1%			
One hundred and thirty-two analytes	Multiclass veterinary drugs	MSPE	HPLC-MS/MS	Milk	LOD: 0.015–0.3 μg/kg	OCT: 1.5 μg/kg, CAP: 4.1 μg/kg, SMZ, LIN: 5.6 μg/kg CIP: 12.2 μg/kg	Russia	[135]
					LOQ: 0.05–1 μg/kg			
					R^2^: <0.990			
					R: 72–120%			
					RSD: <20%			
Sixty-six analytes	Multiclass veterinary drugs	d-SPE and SPE	UHPLC-MS/MS	Cow milk	LOQ: 0.02–18.25 μg/kg	Danofloxacin 0.7–1.5 μg/kg	Spain	[136]
					CCα: 0.01–150.07 μg/kg			
					CCβ: 0.04–150.14 μg/kg			
					R^2^: >0.998			
					R: 70–120%			
					RSD: ≤19.4%			
Fifty-seven analytes	Multiclass veterinary drugs	Modified QuEChERS	UPLC-MS/MS	Milk	LOD: 0.1~3.8 μg/kg	Flumequine and pipemidic	China	[137]
					LOQ: 0.2~6.3 μg/kg			
					LR: 2~500 μg/kg R^2^: ≥0.999			
					R: 60.7–116.0%			
Sixteen analytes	Multiclass veterinary drugs	d-SPE & LLE	LC–MS/MS	Bovine and caprine milk	CCα: 0.023–<5.0 μg/kg	Blank samples are spiked	Netherlands	[26]
					CCβ: 0.045–5.0 μg/kg			
					LR: 5–250 μg/L R^2^: ≥0.990			
Eighteen analytes	Multiclass veterinary drugs	Modified QuEChERS	UHPLC-HR-Orbitrap-MS	Milk	LOD: 0.09–15.1 μg/kg	Imidocarb: 18 μg/kg (one sample)	Greece	[85]
					LOQ: 0.28–10 μg/kg			
					R^2^: >0.9903			
					R: 65.1–120.1%			

LOD, limit of detection; LOQ, limit of quantification; LR, linear range; R^2^, determination coefficient; R, recovery; RSD%, relative standard deviation; CCα, decision limit; CCβ, detection capability; CV, coefficient of variation; ND, not detected; NS, not specified; MDL, method detection limit; MQL, method quantification limit; SPE, solid-phase extraction; MSPE, magnetic solid-phase extraction; FPSE, fabric-phase sorptive extraction; LLE, liquid–liquid extraction; d-SPE, dispersive solid-phase extraction; D-m-SPE, dispersive micro-solid-phase extraction; EME, electromembrane microextraction; CPME, capsule phase microextraction; DLLME, dispersive liquid–liquid microextraction; SPME, solid-phase microextraction; SALLE, salting-out-assisted liquid–liquid extraction; CSMISPE, core–shell molecularly imprinted solid-phase extraction, SMISPE, surface molecularly imprinted solid-phase extraction; SS-DMNF-ME, syringe-to-syringe dispersive magnetic nanofluid microextraction; BSASLE + BUASLE, batch-stirring-assisted solid-to-liquid extraction and batch ultrasound-assisted solid-to-liquid extraction; UA-HDES-DLLME, ultrasound-assisted hydrophobic deep eutectic solvent-dispersive liquid–liquid microextraction; BSE, bar sorptive extraction; MIL-based AALLME, magnetic ionic liquid-based air-assisted dispersive liquid–liquid microextraction; TCs, tetracyclines; SAs, sulfonamides; Qs, quinolones; non-steroidal anti-inflammatory drugs (NASIDs); CLC, capillary liquid chromatography; MLC, micellar liquid chromatography.

**Table 2:** Overview of the analytical methods for the extraction and determination of EDCs residues in dairy milk.

Target EDCs	Extraction Method	Analysis Technique	Matrix	Analytical Parameters	Conc. in Real Samples	Country	Ref.
Bisphenol A (BPA), bisphenol BP (BPBP), bisphenol C (BPC), bisphenol F (BPF), bisphenol FL (BPFL), bisphenol G (BPG), bisphenol M (BPM), bisphenol S (BPS), bisphenol Z (PBZ), bisphenol A diglycidyl ether (BADGE), bisohenol A (2,3-dihydrox-ypropyl) glycidyl ether (BADGE⋅H_2_O), bisphenol A bis (2,3-dihydrox-ypropyl) ether (BADGE⋅2 H_2_O), bisphenol A (3-chloro-2-hydroxypropyl) glycidyl ether (BADGE⋅HCl), bisphenol A (3-chloro-2hydroxypropyl) (2,3-dihydroxypropyl) ether (BADGE⋅H_2_O⋅HCl), bisphenol A bis(3-chloro-2-hydroxypropyl) ether (BADGE⋅2HCl), bisphenol F diglycidyl ether (BFDGE), bisphenol F bis(2,3-dihydroxypropyl) ether (BFDGE⋅2 H_2_O), bisphenol F bis (3-chloro-2-hydroxypropyl) ether (BFDGE⋅2HCl)	UA–solvent extraction of porous membrane-packed samples	HPLC–MS/MS	Infants’ and toddlers’ ready-to-feed milk and powdered milk	LOD: 0.24–0.40 ng/g	0.53–18.5 ng/g	Poland	[36]
				LOQ: 0.72–1.2 ng/g			
				LR: 1–50 ng/mL R^2^: >0.9962			
				R: 31–120%			
				RSD: 0.3–10%			
BPA, BPAF, BPC, BADGE, BFDGE	Online SPE	HPLC-FLD	Cow and goat milk	LOD: 1.5–2.25 μg/kg	NS	Czech Republic	[143]
				LOQ: 5–7.5 μg/kg			
				LR: 2.5–100 μg/kg			
				R: 93.0–139.2%			
				RSD: <10%			
BPA	SPE	HPLC-DAD	Bovine milk	LOD: 1.3 ng/mL	Spiked	China	[149]
				LR: 0.02–2 mg/mL R^2^: 0.9998			
				R: 96.4–102.8%			
				RSD: 1.5–6.3%			
BBA	SPE	LC-FLD	Cow-milk-filled plastic baby bottles from different brands	LOD: 3.75 ng/mL LOQ: 12.51 ng/mL LR: 40.0–120.0 ng/mL R^2^: 0.9970 R: 83–88% RSD%: 2.21%, 9.55%	BPA: <LOQ–102.18 ng/mL	Italy	[65]
BPS		LC-UV		LOD: 80.00 ng/L LOQ: 260.00 ng/mL LR: 1.0–3.0 μg/mL R^2^: 0.9989 R: 95–108% RSD: 1.81%, 5.03%	ND		
BPA, BADGE, BPAF, BPAP, BPB, BPBP, BPC, BPE, BPF, BFDGE, BPM, BPP, BPZ, 4-octylphenol (4-OP) 4-tert-octylphenol (4-t-OP) 4-nonylphenol (4-NP)	d-SPE + QuEChERS	HPLC–FLD	Raw buffalo milk and retail bovine milk	LOD: 0.2, 0.6 ng/g	Raw buffalo milk: 4-t-OP: 1.41 ng/g BFDGE: 1.10 and 1.33 ng/g BPF, BPC, and 4-NP: between LODs and LOQs Retail bovine milk: BPA: 1.11–3.05 ng/g BPP, BPM, 4-t-OP, 4-OP: >LOD detected but not quantified	Italy	[33]
				LOQ: 1.0, 3.0 ng/g			
BPA, BADGE, BPAF, BPAP, BPB, BPBP, BPC, BPE, BPF, BFDGE, BPG, BPM, BPP, BPS, BPZ, bisphenol PH (BPPH), bisphenol TMC (BPTMC)	SPE	UHPLC–MS/MS	Raw Buffalo milk and retail bovine milk	LOD: 0.03–1.5 ng/mL	Raw buffalo milk: BPA: 0.5–5.6 ng/mL BPF: 0.5–8.7 ng/mL BPAF: 3.0 ng/mL Retail bovine milk: BPA: ND–2.8 ng/mL BPF: ND–10.6 ng/mL	Italy	[33]
				LOQ: 0.1–5.0 ng /mL			
BPA, BPB, BPAF, BPC	MSPE	HPLC-UV	Milk	LOD: 0.011–0.36 ng/mL	BPA: 0.79–4.56 ng/mL	China	[150]
				LOQ: 0.035–0.120 ng/mL			
				LR: 0.05–100 ng/mL R^2^: 0.9980–0.9998			
				R: 85.70–119.7%			
				RSD: 0.12–5.02%			
BPA, BADGE, BPAF, BPAP, BPB, BPBP, BPC, BPE, BPF, BFDGE, BPG, BPM, BPP, BPPH, BPS, BPTMC, and BPZ	SPE	UHPLC-MS/MS	Bovine and buffalo milk	LOD: 0.03–0.6 ng/mL	0.1–2.0 ng/mL	Italy	[33]
				LOQ: 0.1–5.0 ng/mL			
				R^2^: >0.95			
BPA	SPE	HPLC-FLD	Raw cow milk	LOD: 0.01 μg/kg	0.035–2.776 μg/L	Italy	[144]
				LOQ: 0.03 μg/kg			
				LR: 0.03–100 μg/L R^2^: 0.9969			
				R: 70–100%			
				RSD: ≤10%			
BPA	DME	HPLC-FLD	Skimmed milk samples	LOD: 0.016 μg/L	ND	China	[151]
				LOQ: 0.050 μg/L			
				LR: 0.1–50 μg/L R^2^: 0.9964			
				R: 80.7–102.4%			
				RSD: <4.2%			
BPA, BPF, BPAF, 4-CP	UA-DLLME	HPLC-UV	Commercial boxed milk	LOD: 0.25–1 μg/L	ND	China	[152]
				LOQ: 0.5–1 μg/L			
				LR: 0.5–400 μg/L R^2^: 0.9976–0.9988			
				R: 82.77–118.92%			
				RSD: <14%			
BPA	SPE	HPLC-FLD	Milk	LOD: 0.03 μg/L	<LOQ–2.833 μg/L	Italy	[153]
				LOQ: 0.1 μg/L			
				LR: 0.1–100 μg/L R^2^: 0.999			
				R: 78.4–107.2%			
				RSD%: 1.9–11.3%			
Nonylphenol (NP), BPA, hexestrol (HEX)	MSPE	HPLC-UV	Milk	LOD: 0.1–0.3 μg/L	ND	China	[35]
				LR: 0.04~50 mg/L R^2^: 0.9978–0.9992			
				R: 89.9–98.7%			
				RSD: <3%			
BPA, NP, octylphenol (OP), 4-n-nonylphenol (4NP)	QuEChERS	LC-LTQ/Orbitrap MS	Milk	LOD: 0.05–5 ng/g	BPA: MDL-10.4 μg/Kg OP: <4.5 μg/Kg NP & 4NP: <428.7 μg/Kg	Greece	[154]
				LOQ: 0.1–20 ng/g			
				LR: 0.1–200 ng/g R^2^: 0.9966–0.9999			
				R: 91–108%			
				RSD: 0.9–11.7%			
BPA, α-estradiol (α-E2), genic EDCs; 17α-ethinyl estradiol (17α-EE2), estrone (E1), diethylstilboe-strol (DES), and hexestrol (HEX)	FPSE	HPLC-UV & LC-MS/MS for confirmation	Milk	LOD: 7.5–15 ng/mL	All spiked	USA	[14]
				LOQ: 25.0–50.0 ng/mL			
				LR: 25–20,000 ng/mL			
				R: 13.7–69.2%			
				RSD: 3.6–13.9			
BPA	SPE	HPLC-FLD	Raw cow milk	LOD: 0.01 μg/kg	ND–2.340 μg/L	Italy	[155]
				LOQ: 0.03 μg/kg			
				LR: 0.03–100 μg/L			
BPF	SPE	HPLC-FLD	Milk	LOD: 0.03 μg/L	<LOQ–2.956 μg/L	Italy	[156]
				LOQ: 0.1 μg/L			
				LR: 0.1–100 μg/L R^2^: 0.999			
				R: 97.60–107.16%			
				RSD: <15%			
BFDGE·2H_2_O, BADGE·2H_2_O, BFGDGE·H_2_O, BPE, BPA, BPB, BPC, para-para-BFDGE, BADGE	QuEChERS	HPLC–FLD	Milk	LOD: 1.0–3.1 μg/kg	BPA: 13.74 μg/ kg (one sample) BADGE·2H_2_O: 15.80 μg/kg (one sample) BFDGE·2H_2_O: 16.23 and 17.82 μg/kg	China	[86]
				LOQ: 3.5–9.8 μg/kg			
				LR: 5–100 μg/kg R^2^: 0.9942–0.9997			
				R: 75.82–93.86%			
				RSD: 2.6–11.1%			
BPF	SPE	HPLC-FLD	Milk	LOD: 0.03 μg/L	<LOQ–2.686 μg/L	Italy	[153]
				LOQ: 0.1 μg/L			
				LR: 0.1–100 μg/L R^2^: 0.999			
				R: 97.60–107.16%			
				RSD: <15%			
Methylparaben (Me-P), ethylparaben (Et-P), propyl-paraben (Pr-P), butylparaben (BP), benzylparaben (BzP), BPA, BPS, BPF, BPB, BPE, BPAF	QuEChERS +d-SPE	HPLC-MS/MS	Raw and processed cow milk	LOD: 0.01–0.2 ng/mL	Bisphenols: <LOD–1.71 ng/mL Parabens: <LOD–1.40 ng/mL	Poland	[146]
				LOQ: 0.03–0.73 ng/mL			
				LR: 0.5–2000 ng/mL R^2^: 0.9988–0.9997			
				R: 80.1–115.5%			
				RSD: 1.8–9.4%			
Me-P, Et-P, Pr-P	SC-μSPE	HPLC-UV	Milk	LOD: 3.0–7.0 ng/mL	<LOQ–130.3 ng /mL	Iran	[148]
				LOQ: 10–20 ng/mL			
				LR: 10–1000 ng/mL R^2^: 0.9960–0.9971			
				R: 81.7–97.8%			
				RSD: 2.7–8.6%			
Estrone E1, 17β-estradiol (E2), estriol E3, and BPA	MSPE	HPLC-MS/MS	Cow milk	LOD: 0.37–0.85 μg/L	ND	China	[157]
				LOQ: 1.31–2.94 μg/L			
				LR: 0.25–100 μg/L R^2^: ≥0.9983			
				R: 92.1–118.3%			
				RSD: ≤7.2%			
BBP, benzyl butyl phthalate; DEHP, bis (2-ethylhexyl) phthalate; DIDP, diisodecyl phthalate; DIHP, diisoheptyl phthalate; DNOP, di-n-octyl phthalate; DPP, dipentyl phthalate.	MSPE	GC-MS/MS	Milk	LOD: 0.8–2.1 μg/L	ND	China	[60]
				LOQ: 2.7–7.0 μg/L			
				LR: 3.0–100 μg/L			
				R: 76.8–99.2%			
				RSD: ≤7.3%			
BBP, butyl benzyl phthalate; BPA, bisphenol A; DBP, dibutyl-o-phthalate, DEHP, di(2-ethylhexyl) phathalate; DEP, diethyl-o-phthalate; DNOP, di-n-octyl phthalate	PFSPE	GC-MS	Milk	LOD: 0.01–0.06 μg/L	DEP: ND–2.18 μg/L DBP: ND–1.5 μg/L BPA: 0.28–2 μg/L BBP: 10.98–16.0 μg/L DEHP: ND–16.20 μg/L DNOP: 0.27–0.50 μg/L	China	[59]
				LOQ: 0.05–0.53 μg/L			
				LR: 0.1–50 μg/L R^2^: 0.9925–0.9987			
				R: 89.6–118.0%			
				RSD: 0.6–10.9%			
Phenol, 2,5-dimethylphenol, 4-chlorophenol, 3,4-dimethylphenol, 4-chloro-3-methylphenol, 4-tert-butylphenol, 2-tert-butyl-4-methylphenol, 4-pentylphenol, 2-phenylphenol, 4-hexylphenol, 4-tert-octylphenol, 4-heptylphenol, nonylphenol, 4-phenylphenol, pentachlorophenol, triclosan, bisphenol F, bisphenol A, bisphenol B, bisphenol Z, bisphenol S	SPE	GC-MS	Cow, goat, and sheep milk	LOD: 6–35 ng/kg	BPA: 30–940 ng/kg BPZ: 96–1100 ng/kg BPF: 270–950 ng/kg NP: 58–390 ng/kg 4-t-BP: 310–2100 ng/kg 3,4-DMP: 130–1800 ng/kg	Spain	[158]
				LR: 20−10 000 ng/kg R^2^: 0.994–0.999			
				R: 86–106%			
2-chlorophenol, o-cresol m-cresol, 2,4-dichlorophenol, 4-tert-butylphenol, 4-chlorophenol, 4-tertoctylphenol, alpha-naphthol	EA–SPME	GC–FID	Milk	LOD: 0.001–0.1 μg/L	ND–31.07 μg/L	China	[57]
				LOQ: 0.1 μg/L			
				LR: 0.005–50 μg/L R^2^: >0.99			
				R: 87.3–118.9%			
				RSD: 1.9–12.3%			
Metylparaben, ethyl-paraben, propylparaben, isopropylparaben, butylparaben, isobutylparaben, benzyl-paraben, dichlovos, dimethoate, diazinon, bromophos methyl, chloropyrifos, fenthion, fenthion sulphoxide, parathion methyl, malathion, methidathion, nonylphenol, 4-tert-ocylphenol, 2-phenylphenol, 4-phenylphenol, BPA and triclosan (TCS)	SPE	GC-MS	Cow, sheep and goat milk	LOD: 6–40 ng/kg	ethylparaben 120–3100 ng/kg 2-phenylphenol: 130–2000 ng/kg BPA: 980–4600 ng/kg 4-Phenylphenol: 130–230 ng/kg Butylparaben: 620 ng/kg	Spain	[159]
				LR: 20–10,000 ng/kg			
				R: 80–107%			
				RSD: 2.6–7.1%			
Mep, EtP, *n*-Prp, propyl 4-hydroxybenzoate; *n*-Bup, butylparaben; i-Prp, isopropyl 4-hydroxybenzoate; i-BuP, isobutylparaben	MSPE	GC–MS	Milk	LOD: 0.1 ng/mL	NS	China	[160]
				LOQ: 0.5 ng/mL			
				LR: 0.1–600 ng/mL R^2^: 0.9991–0.9997			
				R: 95–105%			
				RSD: 2.7–5.0%			

LOD, limit of detection; LOQ, limit of quantification; LR, linear range; R^2,^ determination coefficient; R, recovery; RSD%, relative standard deviation; CCα, decision limit; CCβ, detection capability; CV, coefficient of variation; ND, not detected; NS, not specified; UA, ultrasound-assisted; SPE, solid-phase extraction; dSPE, dispersive solid-phase extraction; MSPE, magnetic solid-phase extraction; DME, dispersive-membrane solid-phase extraction; UA-DLLME, ultrasound-assisted dispersive liquid–liquid microextraction; FPSE, fabric-phase sorptive extraction; SC-μSPE, spin-column micro-solid-phase extraction; PFSPE, packed-nanofiber solid-phase extraction; EA-SPME, electrochemical assistance solid-phase microextraction.

**Table 3:** Overview of the analytical methods for the extraction and determination of pesticides residues in dairy milk.

Target Pesticides	Extraction Method	Analysis Technique	Matrix	Analytical Parameters	Conc. in Real Samples	Country	Ref.
Lindane, alachlor, aldrin, bromophos methyl, heptachlor epoxide, α-endosulfan, hexaconazole, dieldrin, endrin, β-endosulfan, diazinon, endosulfan-sulfate, bromopropylate, fenpropathrin, tetradifon, fenvalerate	QuEChERS-TA-SFOD	GC-μECD	Pasteurized bovine milk	LOD: 0.01–0.11 μg/kg	1.24–4.68 μg/kg	Iran	[161]
				LOQ: 0.03–0.38 μg/kg			
				LR: 0.03–250 μg/kg			
				R: 61–119%			
				RSD: 2.1–18.2%			
Acetamiprid, azinphos-methyl, azoxystrobin, benalaxyl, boscalid, bupirimate, carbaryl, carbendazim, cymoxanil, cyprodinil, dichlorvos, dimethoate, fenthion sulfoxide, imidacloprid, iprovalicarb, metalaxyl, myclobutanil, tebuconazole, thiacloprid, thiamethoxam	Modified QuEChERS	UHPLC-LTQ/Orbitrap MS	Full-fat cow and goat milk	LOD: 0.2–8.1 μg/kg	Carbendazim < LOQ one sample	Greece	[164]
				LOQ: 0.61–24.8 μg/kg			
				LR: 1–250 μg/kg R^2^: ≥0.9918			
				R: 79.5–119.5%			
				RSD: ≤11.7%			
Imidacloprid, acetamiprid, nitenpyram, thiacloprid	DSPE–SFOD–DLLME	HPLC–DAD	Pasteurized semi-skimmed cow milk	LOD: 0.13–0.21 ng/mL	All samples are spiked	Iran	[162]
				LOQ: 0.43–0.70 ng/mL			
				LR: 0.70–500 ng/mL			
				R: 73–85%			
				RSD: 1.4–5.1			
One hundred and ninety-five pesticides	Modified QuEChERS	LC-Q-TOF/MS	Raw milk	Screening detection limits (SDL): 0.1–20 μg/kg	ND	China	[31]
				LOQ: 0.1–50 μg/kg			
				LR: 1–200 μg/kg R^2^: >0.99			
				R: 70.0–120.0%			
				RSD: <20			
Dimethoate, imidacloprid, pirimicarb, carbaryl, fenitrothion, hexythiazox, phosalone	OPD-SPME-DES	HPLC-MS/MS	Pasteurized cow milk	LOD: 0.09–0.27 ng/mL	ND	Iran	[41]
				LOQ: 0.31–0.93 ng/mL			
				LR: 0.93–500 ng/mL			
				R: 81–94%			
				RSD: <9%			
Imidacloprid, thiamethoxam, thiacloprid, clothianidin, acetamiprid	SPE	LC–MS/MS	Sheep and cow milk	LOD: 0.5 μg/kg	ND	Jordan	[165]
				LOQ: 1 μg/kg			
				LR: 1–100 μg/kg R^2^: >0.999			
				R: 75.1–88.3%			
				RSD: 4.3–31.2%			
Azinphos-methyl, parathion-methyl, phosalone, diazinon, chloropyrifos	DSPE–DLLME	HPLC–DAD	Milk	LOD: 0.17–0.36 ng/mL	Chloropyrifos in one sample: 19 ± 0.8 ng/mL	Iran	[166]
				LOQ: 0.57–1.34 ng/mL			
				LR: 1.34–1000 ng/mL R^2^: 0.992–0.996			
				R: 79–92%			
				RSD: ≤7.2%			
Metolcarb, carbaryl, isoprocarb, bassa, diethofencarb	SPE	HPLC-DAD	Milk	LOD: 0.12–0.40 ng/mL	ND	China	[167]
				LOQ: 0.36–1.20 ng/mL			
				LR: 1.0–320.0 ng/mL			
				R: 86.0 to 110.0%			
				RSD: 4.9–6.3			
Spinosyn A and D, temephos, piperonyl butoxide	LLE followed by QuEChERS	LC-MS/MS	Milk	LOD: 0.1–1.4 μg/kg	ND	Korea	[168]
				LOQ: 0.3–4.1 μg/L			
				LR: 1.5–50 μg/kg R^2^: 0.983–0.996			
				R: 78–99%			
				RSD: <8%			
Tebufenozide (TEB) and indoxacarb (IND)	LLE	LC-MS/MS	Milk	LOD: 5, 1 μg/kg	ND	Korea	[79]
				LOQ: 10, 3 μg/kg			
				LR: 5–50 μg/kg R^2^: 0.998–0.9993			
				R: 87.79–114.93%			
				RSD: <6.4%			
α-HCH, HCB, β-HCH, lindane, δ-HCH, chlorthalonil, heptachlor, aldrin, chlorpyrifos, bromophos, α-endosulfan, dieldrin, p,p′-DDE, p,p′-DDD, p,p′-DDT	Modified QuEChERS	GC-ECD	Cow milk	LOD: 0.00015–0.0009 mg/kg	-	Iran	[37]
				LOQ: 0.0005–0.003 mg/kg			
				LR: 0.0005–0.5 mg/kg R^2^: 0.9943–0.9995			
				R: 65–118%			
				RSD: 1–15%			
Carbendazim, thiabendazole, dichlorvos, carbofuran, dimethoate, carboxin, pirimicarb, terbutryn, thiacloprid, imidacloprid, trichlorfon, fenitrothion, fenthion, cyproconazole, thiamethoxam, tridemorph, fenamiphos, diazinon, pirimiphos-methyl, tebuconazole, butachlor, fenamidone, kresoxim-methyl, sulfotep, diniconazole, malathion, bitertanol, propiconazole, thiophanate-methyl, clodinafop-propargyl, flamprop-isopropyl, phosalone, ethion, dimethomorph, nicosulfuron	Modified QuEChERS	UHPLC-MS/MS	Cow milk	LOD: 0.0003–0.03 mg/kg	Dimethoate in raw milk: 0.045 mg/kg	Iran	[37]
				LOQ: 0.001–0.05 mg/kg			
				LR: 0.001–0.5 mg/kg R^2^: 0.9830–0.9993			
				R: 74–121%			
				RSD: 1–17%			
One hundred and fifty-six pesticide residues	Modified QuEChERS	LC–MS/MS	Milk	LOD: 0.11–2.70 μg/kg	ND	Turkey	[169]
				LOQ: 0.38–8.10 μg/kg			
				LR: 5–100 μg/kg R^2^: ≥0.99			
				R: 70.38–116.40%			
				RSD: <19%			
Sulfoxaflor	Modified QuEChERS	LC-MS/MS	Milk	LOD: 1.8 μg/kg	<LOQ	China	[170]
				LOQ: 5.0 μg/kg			
				R^2^: 0.9990			
				R: 81.1–95.0%			
				RSD: 2.3–11.2%			
Coumaphos, phosmet, fonofos, parathion, pyridaphenthion, phosalone, temephos, profenofos, terbufos, phenthoate, ethion, tetrachlorvinphos, isazophos, pirimiphos-ethyl, fenthion, phoxim, methidathion, triazophos, pirimiphos-methyl, dichlofenthion	MSPE	LC-MS/MS	Fatty whole milk	LOD: 0.001–0.01 μg/L	Pirimiphos-methyl: 0.23 μg/L) (One sample)	China	[171]
				LOQ: 0.2–0.5 μg/L			
				LR: 0.2–250 μg/L R^2^: 0.9978–0.9999			
				R: 0.0–105%			
				RSD: <12.3%			
Carbofuran, carbaryl, propoxur, aminocarb, phenmedipham, ethiofencarb, desmedipham, fenoxycarb, pirimicarb, bendiocarb, methiocarb	LLE	UHPLC-MS/MS	Camel milk	LOD: 0.01 μg/kg	0.345–9.509 μg/kg	UAE	[163]
				LOQ: 0.03–0.04 μg/kg			
				LR: 0.00001–0.5 mg/kg R^2^: 0.9982–1.0000			
				R: 88–103%			
				RSD: ≤5%			
Lindane, diazinon, fenitrothion, malathion, aldrin, α-endosulfan, β-endosulfan, methoxychlor	DLLME	GC-MS	Bovine milk	LOD: 0.90–5.00 ng/mL	ND	India	[81]
				LOQ: 2.5–15 ng/mL			
				LR: 2–1000 ng/mL R^2^: 0.995–0.999			
				R: 86.15–112.45%			
				RSD: 1.06–2.20%			
Endrin and δ-keto endrin	Modified QuEChERS	GC-μECD	Milk	LOD: 0.003 mg/kg	ND	Korea	[61]
				LOQ: 0.01 mg/kg			
				R^2^: 0.9979, 0.9966			
				R: 84.27–105.29%			
				RSD: 2.12–7.59%			
Forty-one multiclass pesticides	QuEChERS	GC-ECD followed by GC-MS	Commercial liquid milk	LOD: 0.001–0.02 μg/mL	Below the LOQ	India	[16]
				LOQ: 0.002–0.05 μg/mL			
				LR: 0.002–1 μg/mL R^2^: >0.98			
				R: 91.38–117.56%			
				RSD: <2.79%			
Permethrin (Perm), deltamethrin (Del), and cypermethrin (Cyp)	USA-MNF-LPME	GC-MS	Cow milk	LOD: 2.8, 2.7 and 2.0 ng/mL	Per: 18.0 ng/L Del: 25.0 ng/L Cyp: 48.0 ng/L	Iran	[172]
				LOQ: 9.43, 8.95, and 6.47 ng/L			
				LR: 0.01–250 μg/L R^2^: 0.9991, 0.9995			
				R: 91.0–105%			
				RSD: 3.5, 3.2, 2.8%			
Chlorpyriphos, malathion, disulfoton, pirimiphos	d-SPE	GC-MS	Commercial bovine milk	LOD: 0.36–0.95 μg/L	ND	Brazil	[58]
				LOQ: 5.0 μg/L			
				LR: 5.0–40.0 μg/L R^2^: 0.9902–0.9963			
				RSD: <19.9%			
α-HCH; β-HCH; γ-HCH; δ-HCH; heptachlor; aldrin; heptachlor epoxide; trans-chlordane; α-endosulfan; cis-chlordane; p.p’-DDE; endrin; β-endosulfan; endosulfan sulfate; p.p’-DDT; endrin ketone; methoxychlor; phthalic acid and p,p’-DDD.	QuEChERS	GC-MS/MS	Cow milk	LOD: 0.011–0.034 μg/kg	p,p-DDE: 0.09 μg/kg p,p-DDT: 0.07 μg/kg	Bangladesh	[173]
				LOQ: 0.049–0.087 μg/kg			
				LR: 5–200 ppb R^2^: 0.92–0.99			
				R: 79.23–98.65%			
α- and β-hexachlorocyclohexane, lindane, hexachlorobenzene, p,p′-DDE, aldrin, dieldrin, and α-endosulfan	GDME	GC-ECD & GC-MS	Milk	LOD: 3.7 to 4.8 μg/L	Aldrin was found in one sample below the LOD	Brazil	[92]
				LOQ: 12–16 μg/L			
				R^2^: 0.991–0.995			
				R: 71–99%			
				RSD: <10%			
Alpha-cypermethrin,beta-cyfluthrin, bifenthrin, bromopropylate, chlorothalonil, chlorpropham, deltamethrin, dicofol, endosulfan alpha, endosulfan beta, endosulfan sulfate, fenitrothion, fenthion, fenvalerate, formothion, kresoxim methyl, lambda cyhalothrin, oxyfluorfen, permethrin, procymidone, prothiofos, tau-fluvalinate, tetradifon, trifluralin, vinclozolin	QuEChERS	GC–MS	Milk	LOD: 0.31–1.91 μg/kg	ND	Turkey	[174]
				LOQ: 1.05–6.62 μg/kg			
				LR: 5–100 μg/kg R^2^: >0.99			
				R: 72.50–119.54%			
				RSD: 1.17–14.62%			
Linden, heptachlor, aldrin, dieldrin, endrin, endosulfan, dichlorodiphenyltrichloroethane (DDT)	QuECheRS	GC-ECD	Organic and conventional goat milk	LOD: 0.3 ppb	ND	Indonesia	[175]
Dichlorvos, carbaryl, atrazine, ametryne, diazinon, pirimiphos-methyl, carbofuran, chlorpyrifos, prothioconazole, tebuconazole	QuChERS-DLLME	GC-FID	Milk	LOD: 4.2–27.4 ng/mL	Dichlorvos, atrazine, diazinon, chlorpyrifos and tebuconazole 2.49–10.48 ng/mL	Iran	[176]
				LOQ: 11.89–82.23 ng/mL			
				LR: 0.5–100 ng/mL			
				R: 77.69–147.69%			
				RSD: 1.6–9.7%			
Carbaryl, hexythiazox, pretilachlor, iprodione, famoxadone, sethoxydim, fenazaquin	In matrix-DES-SFO-DLLME	GC-FID	Cow milk	LOD: 0.90–3.9 ng/mL	ND	Iran	[177]
				LOQ: 3.1–13 ng/mL			
				LR: 4.5–5000 ng/mL			
				R: 64–89%			
				RSD: 3.8–5.3%			

LOD, limit of detection; LOQ, limit of quantification; LR, linear range; R^2^, determination coefficient; R, recovery; RSD%, relative standard deviation; CCα, decision limit; CCβ, detection capability; CV, coefficient of variation; ND, not detected; NS, not specified; SPE, solid-phase extraction; MSPE, magnetic solid-phase extraction; LLE, liquid–liquid extraction; dSPE, dispersive solid-phase extraction; DLLME, dispersive liquid–liquid microextraction; QuEChERS-TA-SFOD, QuEChERS-temperature-assisted solidification of floating organic droplet; OPD-SPME-DES, organic polymer-based dispersive solid-phase-microextraction deep eutectic solvent; USA-MNF-LPME, ultrasound-assisted magnetic nanofluid-based liquid-phase microextraction; GDME, gas-diffusion microextraction.

**Table 4:** Overview of the analytical methods for the extraction and determination of mycotoxins residues in dairy milk.

Target Mycotoxins	Extraction Method	Analysis Technique	Matrix	Analytical Parameters	Conc. in Real Samples	Country	Ref.
Aflatoxin B_1_ (AFB_1_), aflatoxin B_2_ (AFB_2_), aflatoxin G_1_ (AFG_1_), aflatoxin G_2_ (AFG_2_), aflatoxin M_1_ (AFM_1_), alternariol methyl ether (AME), alternariol (AOH), beauvericin (BEA), cyclopiazonic Acid (CTA), citrinin (CTN), diacetoxyscirpenol (DAS), deepoxy-deoxynivalenol (DOM-1), deoxynivalenol (DON), 15 acetyl-deoxynivalenol (15 AC-DON), 3 acetyl-deoxynivalenol (3 AC-DON), enniatin A (ENNA), enniatin A1 (ENNA1), enniatin B (ENNB), enniatin B1 (ENNB1), fusaric acid (FA), fumonisin B_1_ (FB_1_), fumonisin B_2_ (FB_2_), HT-2 toxin (HT-2), hydrolyzed fumonisin B_1_ (Hydro-FB_1_), mycophenolic acid (MPA), neosolaniol (NEO), ochratoxin A (OTA), roquefortine C (RC), sterigmatocystin (STC), T-2 toxin (T-2), zearalenone (ZEN), zearalanone (ZOL), α-zearalenol (α-ZEN), α-zearalanol (α-ZOL), β-zearalenol (β-ZEN), β-zearalanol (β-ZOL), deoxynivalenol-3-glucoside (DON-3-Gluc), fusarenon X (FX), patulin (PAT), T-2 triol	QuEChERS	UHPLC-MS/MS	Raw milk	LOD: 0.001–3.26 μg/L	T-2, RC, ENNA, ENNA_1_, ENNB, ENNB_1_ and BEA: <LOD–4.76 µg/L	Portugal	[192]
				LOQ: 0.002–10.76 μg/L			
				LR: 0.002–200 μg/L			
				R: 61.22–120.63%			
				RSD: <16%			
AFB_1_, AFB_2_, AFG_1_, AFG_2_, AFM_1_, AFM_2_	IAC	HPLC-MS/MS	Milk	LOD: 0.005–0.010 μg/L	AFM_1_: 0.072 μg/L (one sample)	China	[193]
				LOQ: 0.010–0.026 μg/L			
				LR: 0.010–10.0 μg/L R^2^: 0.988–0.997			
				R: 85.5–106.2%			
				RSD: <12.5%			
AFM1	IAC	HPLC-FLD	Pasteurized cow milk gathered during different seasons	LOD: 0.0001 μg/L	0.002–0.09 μg/L	Iran	[194]
				LOQ: 0.0005 μg/L			
				R^2^: >0.999			
AFM_1_	AALLME	HPLC–FLD	Unpasteurized milk	LOD: 0.9 ng/L	46–96 ng/L	Iran	[83]
				LOQ: 3 ng/L			
				LR: 3–3000 3 ng/L R^2^: 0.9976			
				R: 87 ± 4%			
				RSD: ≤9%			
OTA, AFM_1_	DSPE-DLLME-SFO	HPLC-FLD	Raw cow milk	LOD: 0.25, 0.37 ng/L	OCT A: 35–43 ng/L AFM1: 15–182 ng/L	Iran	[45]
				LOQ: 0.83, 1.23 ng/L			
				LR: 0.83–10^5^, 1.23–10^5^ R^2^: 0.998, 0.997			
				R: 87, 75%			
				RSD: ≤5.1			
OTC, AFB_1_, AFB_2_, AFG_1_, AFG_2_, AFM_1_, AFM_2_, HT-2 Toxin, T-2 Toxin, OTA, DON, OCT α, OCT B, ZEN, α-ZEN, α-ZOL, β-ZEN, β-ZOL, stachybotrylactam, and (S)-zearalanone	QuEChERS	HPLC-MS/MS	cow milk	LOD: 0.007–1.300 μg/kg	<LOD	China	[195]
				LOQ: 0.02–4.00 μg/kg			
				LR: 0.01–10 μg/L R^2^: ≥0.9933			
				R: 80.00–112.50%			
				RSD: 2.67–14.97%			
AFB_1_, AFB_2_, AFM_1_, AFM_2_	ISDμSPE	HPLC-FLD	Cow milk	LOD: 0.003–0.005 ng/mL	AFM_1_: 0.038 ng/mL (One sample)	Malaysia	[76]
				LOQ: 0.01–0.02 ng/mL			
				LR: 0.01–1.0 ng/mL R^2^: 0.992–0.999			
				R: 73.0–109.6%			
				RSD: <17.3%			
AFB_1_, AFM_1_	QuEChERS	UHPLC-Q-Orbitrap HRMS	Milk	LOD: 0.001 μg/L	ND	Italy	[196]
				LOQ: 0.002 μg/L			
				LR: 0.002–20 μg/L R^2^: >0.9990			
				R: 75–96%			
				RSD: <16			
AFM_1_	IAC	LC-FLD	Milk	LOD: 0.01 ng/mL	10–77 ng/L	Morocco	[75]
				LOQ: 0.03 ng/mL			
				R: 87–95%			
				CV: <15%			
AFM_1_, AFB_1_, AFB_2_, AFG_1_, AFG_2_, OTA, OTB, FB_1_, FB_2_, FB_3_, HT-2 and T-2 toxins, nivalenol (NIV), DON, DOM-1, 3 AC-DON, 15 AC-DON, DAS, FX, NEO, STC, and ZEN	LLE	LC–MS/MS	Cow Milk	LOD: 0.010–5.07 ng/mL	OCT A: <LOQ (0.2 ng/mL)	Peru	[186]
				LR: 0.04–101.4 ng/mL R^2^: 0.9935–0.9997			
				R: 61.2–83.9%			
				RSD: 3.8–11.8%			
AFM_1_	IAC	HPLC-FLD	Liquid and powder milk	LOD: 0.002 μg/L	0.021–2.89 μg/L	Yemen	[46]
				R^2^: 0.99995			
				R: 102.94–108.31%			
				RSD: <10%			
AFM_1_	IAC	UPLC-MS/MS	Cow, goat, and sheep milk	LOD: 0.0027 μg/kg	<LOD–0.0370 μg/kg	Greece	[197]
				LOQ: 0.0089 μg/kg			
				LR: 0.75–22.5 μg/L R^2^: 0.997			
				R: 77.9–81.0%			
				RSD: 6.1–12%			
AFB_1_, AFB_2_, AFG_1_, AFG_2_, AFM_1_, AFM_2_, OTA, ZEN, ZOL, α-ZEN, β-ZEN, α-ZOL, β-ZOL	MSPE	UHPLC-Q-Exactive HRMS	Commercial liquid milk	LOD: 0.005–0.050 μg/kg	0.026–0.039 μg/kg	China	[198]
				LOQ: 0.015–0.150 μg/kg			
				LR: 0.15–100 ng/mL R^2^: 0.9963–0.9999			
				R: 81.8–106.4%			
				RSD: 2.1–11.7%			
AFB_1_, AFB_2_, AFG_1_, AFG_2_, OTA, ZEA	IAC	HPLC-FLD	Raw cow milk	LOD: 0.02–0.92 μg/kg	AFM1: <LOQ–0.19 μg/kg	Egypt	[74]
				LOQ: 0.06–2.8 μg/kg			
AFB_1_, AFB_2_, AFG_1_, AFG_2_, AFM_1_, BEA, CTN, DON, ENNA, ENNB, FB_1_, FB_2_; moniliformin (MON); MPA, NIV, OTA, penicillic Acid (PA), PAT, tenuazonic acid (TEA),tentoxin TTX, ZEN.	Modified QuEChERS	UHPLC-MS/MS	Raw cow milk	LOD: 0.001–9.88 ng/mL	NS	Portugal	[199]
				LOQ: 0.005–13.54 ng/mL			
				LR: 0.025–200 ng/mL R^2^: 0.9519–0.9996			
				R: 67.5–119.8%			
				RSD: <25%			
AFM_1_	DLLME	HPLC-FLD	Cow and buffalo milk	LOD: 0.002 μg/L	0.01–9.18 μg/L	India	[200]
				LOQ: 0.007 μg/L			
				LR: 0.01–1.0 μg/L R^2^: 0.999			
				R: 80.9–89.2%			
				RSD: <14%			
AFM_1_, AFM_2_	IAC	HPLC-FLD	Cow, goat, and sheep milk	LOD: 11.99, 16.95 ng/kg	AFM_1_: 47.1–73.4 ng/kg AFM_2_: <LOQ	Greece	[201]
				CCα: 56.52, 57.27 ng/kg			
				CCβ: 63.97, 65.57 ng/kg			
				R^2^: 0.999, 0.996			
				R: 74–120%			
				RSD: <17%			
AFB_1_, AFM_1_, OTA, ZEN, α-ZEN, β-ZEN, ZOL, α-ZOL, β-ZOL	SPE	UHPLC-MS/MS	Milk	LOD: 0.01–0.07 ng/mL	AFM_1_: 0.03–0.30 ng/mL ZEA: 0.3, 1.46 and 2.99 ng/mL	China	[69]
				LOQ: 0.02–0.18 ng/mL			
				LR: 0.02–200 ng/mL R^2^: ≥0.992			
				R: 70.2–111.2%			
				RSD: 2.0–14.9%			
ENNA, ENNA1, ENNB, ENNB1, BEA.	LLE	LC-MS/MS	Cow milk	LOD: 0.088–0.099 μg/kg	ENNB: 0.157–0.587 μg/kg BEA: 0.101–6.17 μg/kg	Poland	[202]
				LOQ: 0.099–0.130 μg/kg			
				LR: 0.15–50 μg/kg			
				R: 72–99%			
				RSD: 3.4–17.5%			
AFM_1_, AFB_1_	QuEChERS	HPLC-FLD	Milk powder	LOD: 0.038, 0.027 μg/kg	AFM_1_: 0.20–1.19 μg/kg	Colombia	[203]
				LOQ: 0.125, 0.083 μg/kg			
				R: 65–110%			
				RSD: <20%			
AFM_1_	IAC	HPLC-FLD	Milk	LOD: 0.01 μg/L	0.016–0.030 μg/kg	Iran	[204]
				LOQ: 0.03 μg/L			
				R^2^: >0.98			
				R: 90.6% (mean)			
				RSD: 5.7%			
AFB_1_, AFB_2_, AFG_1_, AFG_2_, AFM_1_, AFM_2_, FB1, FB2, STE, ZEN.	MSPE	HPLC–MS/MS	Milk	LOD: 0.003–0.442 μg/kg	NS	China	[70]
				LOQ: 0.008–1.219 μg/kg			
				LR: 0.02–200 μg/kg			
				R: 88.3–103.5%			
				RSD: 2.4–6.5%			

LOD, limit of detection; LOQ, limit of quantification; LR, linear range; R^2^, determination coefficient; R, recovery; RSD%, relative standard deviation; CCα, decision limit; CCβ, detection capability; CV, coefficient of variation; ND, not detected; NS, not specified; IAC, immunoaffinity column; SPE, solid-phase extraction; LLE, liquid–liquid extraction; AALLME, air-assisted liquid–liquid microextraction; MSPE, magnetic solid-phase extraction; DLLME, dispersive liquid–liquid microextraction; DSPE-DLLME-SFO, dispersive solid-phase extraction–dispersive liquid–liquid microextraction–solidification of organic drop; ISDμSPE, in-syringe dispersive micro-solid-phase extraction.

**Table 5:** Overview of the analytical methods for the extraction and determination of residues of other EPs including hormones, mycotoxins, PFASs, and multiclass residues in dairy milk.

Target EPs	Category	Extraction Method	Analysis Technique	Matrix	Analytical Parameters	Conc. in Real Samples	Country	Ref.
Perfluorobutanoic acid (PFBA), perfluoropeptanoic acid (PFPeA), perfluorohexanoic acid (PFHxA), perfluoroheptanoic acid (PFHpA), perfluorooctanoic acid (PFOA), perfluorononanoic acid (PFNA), perfluorodecanoic acid (PFDA), perfluoroundecanoic acid (PFUnDA), perfluorododecanoic acid (PFDoDA), perfluorotridecanoic acid (PFTriDA), perfluorotetradecanoic acid (PFTeDA), perfluorobutane sulfonate (PFBS) perfluoropentane sulfonate (PFPeS), perfluorohexane sulfonate (PFHxS, perfluoroheptane sulfonate (PFHpS), perfluorooctane sulfonate (PFOS), perfluoro-4-ethylcyclohexanesulfonate (PFECHS), perfluorononane sulfonate (PFNS), perfluorodecane sulfonate (PFDS), perfluorobutane sulfonamide (FBSA), perfluorooctane sulfonamide (FOSA), N-methylperfluoro-1-octanesulfonamid (N-MeFOSA), N-ethylperfluoro-1-octanesulfonamide (N-EtFOSA), 4:2 fluorotelomer sulfonate (4:2 FtS), 6:2 fluorotelomer sulfonate (6:2 FtS), 8:2 fluorotelomer sulfonate (8:2 FtS)	PFAS	SLE	HPLC-MS/MS	Cow milk	LOD: 0.8–22 ng/L (PFBA: 144 ng/L)	PFCA, PFSA, PASF: <MDL FTS < MDL–6.59 ng/L	USA	[220]
					R: 70–141%			
PFBA, PFPeA, PFBS, PFHxA, PFHpA, PFOA, PFHxS, PFNA, PFOS, PFDA, PFUdA, PFDS, PFDoA, PFTrDA, and PFTeDA	PFAS	QuEChERS	UHPLC-MS/MS	Dairy milk and infant formulas	LOD: 0.005–0.05 ng/mL	The Σ15 PFAS in dairy milk: 0.08–15.51 ng/mL The Σ15 PFAS in infant formula: 0.01–5.24 ng/mL	South Africa	[42]
					LOQ: 0.005–0.05 ng/mL			
					R^2^: 0.987–0.999			
					R: 93–120%			
					RSD: 3–18%			
PFBA, PFPeA, PFHxA, PFHpA, PFOA, PFNA, PFDA, PFUdA, PFDoA, PFTrDA PFTeDA, PFBS, PFHxS, PFOS, PFDS	PFAS	QuEChERS	UHPLC–MS/MS	Dairy milk and infant formula	CCα: 30–50 ng/kg	Infant formulae: <LOQ–259 ng/kg dairy milk: <LOQ–294 ng/kg	South Africa	[221]
					CCβ: 40–100 ng/kg			
					LOQ: 5–50 ng/kg			
					LR: 5–1200 ng/kg R^2^: 0.9843–0.9998			
					R: 60–121%			
					RSD: 5–28%			
PFPA, PFBS, PFHpA, PFOA, PFHpS, PFNA, PFOS, PFDA	PFAS	SPME	UHPLC-MS/MS	Milk and milk powder	LOD: 0.1–0.8 pg/g	ND–4.12 pg/g	China	[222]
					LOQ: 0.4–2.5 pg/g			
					R^2^: ≥0.992			
					R: 89.8–111%			
					RSD: ≤10%			
PFBA, PFPeA, PFHxA, PFHpA, PFOA, PFNA, PFDA, PFUnA, PFDoA, PFBS, PFHxS, PFOS,	PFAS	QuEChERS	LC-MS/MS	Cow milk	LOD: 7.78–16.35 ng/kg	NS	Italy	[223]
					LOQ: ALL: 50 ng/kg GenX and C6O4: 100 ng/kg			
					R: 91.3–121.8%			
					RSD: ≤10.9%			
PFOA, PFOS	PFAS	DFE	LC-MS/MS	Milk	LOD: 0.006–0.022 ng/mL	0.08–2.19 ng/mL	China	[224]
					LOQ: 0.020–0.072 ng/mL			
					LR: 0.05–100 ng/mL R^2^: ≥0.9998			
					R: 94.7–109%			
					RSD: ≤9.5%			
Melamine	Non-protein nitrogen supplement	DLLME	HPLC-UV	Milk	LOD: 63.64 μg/kg	ND	Iran	[49]
					LOQ: 210.03 μg/kg			
					LR: 210.03–1000 μg/kg			
					R^2^: 0.9898			
					R: 72.5–104.0%			
					RSD: <10.2			
Melamine	Non-protein nitrogen supplement	MSPE	UPLC-MS/MS	Milk powder	LOD: 0.00045 mg/kg	0.023 mg/kg (One sample)	China	[225]
					R: 90.3–95.7%			
					RSD: 0.3–4.7%			
Melamine	Non-protein nitrogen supplement	SPE	HPLC-DAD	Milk powder	LOD: 0.006 mg/kg	0.017–0.082 mg/kg	Uruguay	[226]
					LOQ: 0.019 mg/kg			
					R^2^: >0.999			
					R: ≥83.8%			
					RSD: 0.5–9.9%			
Melamine	Non-protein nitrogen supplement	LPME	HPLC-UV	Milk	LOD: 0.03 mg/L	<LOD	Russia	[48]
					LOQ: 0.1 mg/L			
					LR: 0.1–30 mg/L R^2^: 0.994			
					R: 95%			
					RSD: <7%			
Melamine	Non-protein nitrogen supplement	SPE	HPLC-FLD	Milk and infant formula	LOD: 0.005–0.042 μg/mL	0.18–2.90 μg/mL	Turkey	[208]
					LOQ: 0.015–0.126 μg/mL			
					R: 78–103%			
					RSD: ≤1.21%			
Prednisone (PRD), hydrocortisone (HCOR), methylprednisolone (MPRD), dexamethasone (DXM), betamethasone (BEM), prednisone acetate (PRDA), beclomethasone (BCM), fludrocortisone acetate (FCORA), dexamethasone acetate (DXMA), fluocinolone acetonide (FCA), halcinonide (HAL), triamcinolone acetonide acetate (TCAA), fluocinonide (FLC), nandrolone (NAN), methyltestosterone (MTES), testosterone propionate (TESPR), chlormadinone acetate (CHMA), megestrol acetate (MGA), medroxyprogesterone acetate (MXPROA), estrone (E1), 17 α-oestradiol (17α-E2), estriol (E3)	Hormones	SPE	HPLC-MS/MS	Bovine milk	LOD: 0.10–1.20 μg/kg	NAN, MTES, MXPROA TESPR, HCOR, E1, 17α-E2, E3: 0.11–5.79 μg/kg	China	[30]
					LOQ: 0.33–3.96 μg/kg			
					LR: 2.5–500 μg/kg R^2^: 0.9943–0.9998			
					R: 82.6–95.3%			
Estrone (E1), 17β-estradiol (β-E2), 17α-ethynylestradiol (EE), estriol (E3), diethylstilbestrol (DES), levonorgestrel (NOR), norethisterone (NORET), megestrol actetate (MGA), progesterone (PRO), testosterone (TES), boldenone (BOL), nandrolone (NAN), cortisone (COR), prednisone (PRD), prednisolone (PRDNL)	Hormones	FPSE	UHPLC-MS/MS	Cow and goat milk	LOD: 0.012–1.242 ng/mL	ND	Spain	[27]
					LOQ: 0.04–4.14 ng/mL			
					R: 17.91–59.01%			
β -E2, EE, E1, hexestrol (HEX)	Hormones	MSPE	HPLC-VWD-FLD	Milk powder	LOD: 0.5–0.9 μg/kg	ND	China	[227]
					LOQ: 1.5–3 μg/kg			
					R: 75.1–97.2%			
					RSD: ≤14.2			
E3, PRDA, HCOR, DES, E1	Hormones	Online-SPE	HPLC-UV	Cow Milk	LOD: 0.004–0.054 μg/mL	ND	China	[228]
					LOQ: 0.015–0.180 μg/mL			
					R: 70.82–112.90%			
E2, TES, PRO	Hormones	VALLME-MSPE	HPLC-DAD	Milk	LOD: 1.0–1.3 ng/mL	0.2–4.6 ng/mL	China	[229]
					LOQ: 2.5–4.5 ng/mL			
					R 80.1–116.4%			
					RSD: ≤13.9%			
Progesterone (PRO), trenbolone (TRB), norethisterone (NORET), gestodene (GSD), altrenogest (ALT), dienogestrel (DNG), norgestrel (NOG), demegestone (DMG), 17α-hydoxy progesterone (17 α-HPRO), 21α-hydoxy progesterone (21 α-HPRO), megestrol (MEG), medroxyprogesterone (MXPRO), melengestrol (MLG), chlormadinone (ChMD), drospirenone (DROS), cyproterone (CYP), norethindrone acetate (NORA), megestrol acetate (MGA), medroxyprogesterone acetate (MXPROA), melengestrol acetate (MLGA), chlormadinone acetate (ChMDA) and cyproterone acetate (CYPA)	Hormones	SPE	UHPLC-QE HF HRMS	Cow and ewe milk	LOD: 0.05–0.3 μg /kg	PRO: 0.48–54.2 μg/kg NOG: 1.45 ± 0.21 μg/kg GSD: 3.1 μg/kg MXPROA: 8.05, 152 μg/kg MXPRO: 13.5 μg/kg CYP: 61.2 ± 2.7 μg/kg	China	[29]
					LOQ: 0.2–1 μg /kg			
					R^2^: >0.99			
					R: 80.7–108.3%			
					RSD: <15%			
PCB81, PCB153, PCB105, PCB126, PCB157	PCBs	DSPE	GC–MS/MS	Milk	LOD: 0.14–0.57 pg/g	<lOQ–5.27 pg/g	China	[230]
					LOQ: 0.47–1.90 pg/g			
					LR: 0.002–1.000 ng/g R^2^: 0.9995–0.9998			
					R: 82.8–106%			
					RSD: ≤6.6%			
PCB28, PCB52, PCB101, PCB138, PCB153, PCB180, PCB209, napthalene (NA), 2-methylnapthalene (2-MNA), 1-methylnapthalene (1-MNA), acenapthylene (AcNy), acenapthalene (AcNA), fluorene (FLN), phenanthrene (PhN), anthracene (ANT), fluranthene (FLT), pyrene (PY), benzo (A) anthacene (B-A-ANT), chrysene (Chr), benzo (B) fluoranthene (B-B-FLT), benzo (K) fluranthene (B-K-FLT), benzo (A) pyrene (B-A-PY), indeno (1, 2, 3-CD) pyrene (IPY), dibenz (A, H) anthracene (DANT)	PCBs & PAHs	QuEChERS	GC-MS/MS	Cow milk	LOD: PCBs: 0.016–0.031 ng/g PAHs: 0.3, 1.0 ng/g	PCBs: ND–3.35 ± 0.87 ng/g B-A-ANT: 0.5497 ± 0.30 ng/g Chr: 1.077 ± 0.88 ng/g	Bangladesh	[34]
					LOQ: PCBs: 0.059–0.08 ng/g PAHs: 1.0, 4.0 ng/g			
					R: PCBs: 77.53–92.49% PAHs: 67.90–99.76%			
NA, AcNy, AcNA, FLN, PhN, ANT, FlT, PY, B-A-ANT, Chr, B-B-FLT, B-K-FLT, B-A-PY, IPY, DANT, benzo[g,h,i] perylene (BPer)	PAHs	MSPE	GC–MS	Milk and powder milk	LOD: 0.040–0.075 μg/kg	0.48–1.98 μg/kg	Iran	[231]
					LOQ: 0.121–0.227 μg/kg			
					R: 86.1–100.3%			
					RSD: ≤10.1%			
Furan	Toxic heterocyclic compounds	Automated HS-SPME	GC-MS	Milk	LOD: 0.01 ng/g	ND	Korea	[72]
					LOQ: 0.04 ng/g			
					R^2^: 0.9928–0.9990			
					R: 88.93–95.22%			
					RSD%: 0.91–12.81%			
					RSD: ≤4.9			
Formaldehyde	Adulterants and preservatives	Derivatization, protein precipitation, and solvent extraction	MEKC-UV/DAD	Bovine milk	LOD: 15.0 μg/L	<LOD–0.13 ± 0.02 mg/kg	Brazil	[232]
					LOQ: 50.0 μg/L			
					LR: 50.0–1000 μg/L R^2^: >0.99			
					R: 94.2 ± 0.7%			
					RSD: <3.9%			
Formaldehyde	Adulterants and preservatives	Defatting, protein precipitation, and derivatization	UHPLC-MS/MS	Cow, goat and buffalo milk	LOD: 1 ng/mL	134–255 ng/mL	India	[215]
					LOQ: 6.25 ng/mL			
					LR: 3.12–200 ng/mL R^2^: 0.997–0.999			
					R: >95%			
					RSD: 2.84–8.02%			
Fifty-four analytes	Veterinary drugs and mycotoxins	QuEChERS	UHPLC-Q-Orbitrap HRMS	Milk	LOD: 0.001–0.010 ng/g	0.007–4.530 ng/mL	Italy	[28]
					LOQ: 0.005–0.030 ng/mL			
					R: 60–97%			
					RSD: <14%			
Three hundred and sixty-one analytes	Veterinary drugs and pesticides	LLE + dSPE	LC-MS/MS and GC–MS/MS	Bovine milk	LOQ: 0.02–25 ng/g	Vet drugs: 1.2–18.2 ng/g	India	[39]
					R^2^: ≥0.99			
					R: 70–120% for most of the compounds			
Two hundred and nine analytes	Veterinary drugs, mycotoxins and pesticides	QuEChERS	UHPLC-Qtrap-MS	Raw and commercial milk	LOD: 0.01–1 μg/kg	Sulfamethazine: 1.79 μg/kg cloxacillin: 7.12–69.70 μg/kg aflatoxin M1: 0.17, 0.24 μg/kg fipronil sulfone: 0.08 μg/kg imidacloprid: 6.24 μg/kg acetamiprid: 2.36–12.24 μg/kg	China	[13]
					LOQ: 0.05–5 μg/kg			
					R^2^: ≥0.99			
					R: 51.20–129.76%			
					RSD: 0.82–19.76%			
Sixty-nine analytes	Veterinary drugs, mycotoxins and pesticides	Solvent extraction and SPE	LC–MS/MS	Bovine milk	LOD: 0.0036–47.94 μg/L	Sulfadimethoxine: 27.4, 18.2 μg/L enrofloxacin: 25.7 μg/L tetracycline: 30.1 μg/L oxytetracycline: 41.3 μg/L	North Macedonia	[233]
					LOQ: 0.053–59.43 μg/L			
					CCα: 0.062–211.32 μg/L			
					CCβ: 0.080–233.71 μg/L			
					R^2^: >0.99			
					R: 70.83–109%			
					CV: <24%			
Clanobutin, dichlorvos, and naftazone	Pharmaceuticals and pesticides	LPE	LC–MS/MS	Milk	LOD: 0.04, 0.4,0.1 ng/g	ND	Korea	[234]
					LOQ: 0.1,1,0.4 ng/g			
					LR: 5–50 ng/g R^2^: 0.9916, 0.9807, 0.9833			
					R: 77.5–108.2%			
					RSD: 0.9–12.9%			
BPA, E2, DES, CAP	Hormones, EDCs & antibiotics	MSPE	HPLC-UV	Whole milk and skimmed milk	LOD: 0.004–0.106 μg/mL	ND	China	[235]
					LOQ: 0.008–0.209 μg/mL			
					LR: 0.05–5.00 μg/mL			
					R: 88.17–113.46%			
					RSD: 0.002–1.951%			

LOD, limit of detection; LOQ, limit of quantification; LR, linear range; R^2^, determination coefficient; R, recovery; RSD%, relative standard deviation; CCα, decision limit; CCβ, detection capability; CV, coefficient of variation; ND, not detected; NS, not specified; PFASs, perfluoroalkyl and polyfluoroalkyl substances; PCBs, polychlorinated biphenyls; PAHs, polyaromatic hydrocarbons; SLE, solid liquid extraction; SPE, solid-phase extraction; MSPE, magnetic solid-phase extraction; SPME, solid-phase microextraction; FPSE, fabric-phase sorptive extraction; LPE, liquid-phase extraction; d-SPE, dispersive solid-phase extraction; LLE, liquid–liquid extraction; DFE, dispersive filter extraction; DLLME, dispersive liquid–liquid microextraction; LPME, liquid-phase microextraction; HS-SPME, headspace solid-phase microextraction; VALLME, vortex-assisted liquid–liquid microextraction.

## Data Availability

The data presented in this study are available on request from the corresponding author.
